# Long-Chain Gemini Surfactant-Assisted Blade Coating Enables Large-Area Carbon-Based Perovskite Solar Modules with Record Performance

**DOI:** 10.1007/s40820-023-01155-w

**Published:** 2023-07-14

**Authors:** Yumin Ren, Kai Zhang, Zedong Lin, Xiaozhen Wei, Man Xu, Xianzhen Huang, Haining Chen, Shihe Yang

**Affiliations:** 1 Guangdong Provincial Key Lab of Nano-Micro Material Research, School of Chemical Biology and Biotechnology, Shenzhen Graduate School, Peking University, Shenzhen, 518055 People’s Republic of China; 2 Shenzhen Bay Laboratory, Institute of Biomedical Engineering, Shenzhen, 518107 Guangdong People’s Republic of China; 3 School of Materials Science and Engineering, Beihang University, Beijing, 100191 People’s Republic of China

**Keywords:** Long-chain gemini surfactant, Blade-coating, Capillary number, Carbon-based perovskite photovoltaics, All-printable module

## Abstract

Carbon-based perovskite solar cells show great potential owing to their low-cost production and superior stability in ambient air. However, scaling up to high-efficiency carbon-based solar modules hinges on reliable deposition of uniform defect-free perovskite films over large areas, which is an unsettled but urgent issue. In this work, a long-chain gemini surfactant is introduced into perovskite precursor ink to enforce self-assembly into a network structure, considerably enhancing the coverage and smoothness of the perovskite films. The long gemini surfactant plays a distinctively synergistic role in perovskite film construction, crystallization kinetics modulation and defect passivation, leading to a certified record power conversion efficiency of 15.46% with *V*_oc_ of 1.13 V and *J*_sc_ of 22.92 mA cm^−2^ for this type of modules. Importantly, all of the functional layers of the module are printed through a simple and high-speed (300 cm min^−1^) blade coating strategy in ambient atmosphere. These results mark a significant step toward the commercialization of all-printable carbon-based perovskite solar modules.

**Supplementary Information:**

The online version contains supplementary material available at 10.1007/s40820-023-01155-w.

## Highlights

- Trace amounts of long-chain gemini surfactants are essential for blade-coating of high-quality perovskite films, enabling a 17.05% efficient full printed carbon-based module (50 cm^2^ active area).
- Only when the surfactant chain is over a critical length will the gemini surfactant be effective for blade-coating of perovskite films.
- The surfactants increase the capillary number of perovskite precursor solution, reduce the local disturbance and combat inhomogeneous solidification during blade coating, thus allowing high-quality perovskite films to be formed.

**Supplementary Information:**

The online version contains supplementary material available at 10.1007/s40820-023-01155-w.

## Graphical abstract

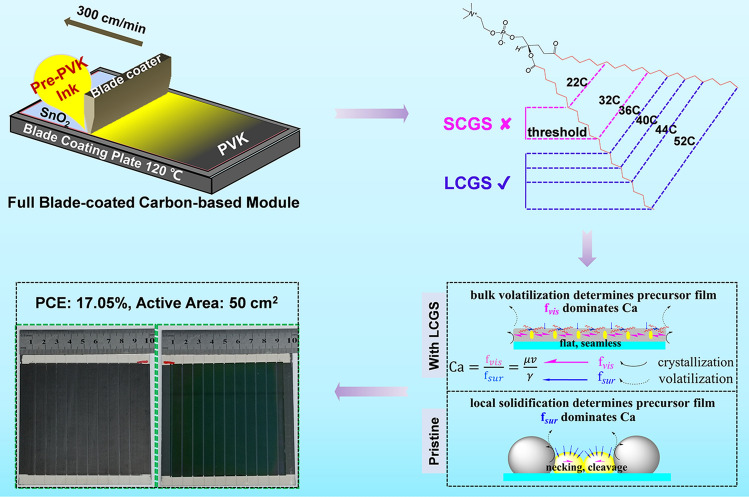

## Introduction

Perovskite solar cells (PSCs) have set off a great wave in the past decade owing to their high-performance, solution processability, low cost and potential for mass production with roll-to-roll printing technologies. Within a very short time span, a certified power conversion efficiency (PCE) of 25.7% has been reported [1]. However, high efficiency PSCs are so far mainly achieved by employing precious metals as electrodes, such as Ag and Au [2–5]. The problem with such metal electrodes is that ion migration widely known in halide perovskite [6, 7] will lead to etching reactions of the migrated halide anions with the metals to form resistive compounds such as AgI [8], jeopardizing the cell efficiency and stability. Although the formation of Au-I is much more difficult, Au atoms could diffuse from electrode into perovskite layer to form anti-site defects of Au_Pb_ with a deep level, which will become efficient non-radiative recombination centers [9].

Carbon-based electrodes represent a green alternative because of their suitable Femi level, hydrophobicity-associated resistance to water and ion-migration, ultralow cost and atmospheric preparation process [10–14]. Thus, carbon-electrode-based PSCs have become one of the most promising competitors in the commercialization of emerging solar cells. However, most efforts have been focused on small-area (< 0.1 cm^2^) PSCs commonly fabricated using the spin-coating method [15, 16]. Scaling-up PSC production has become one of the most pressing challenges in bringing PSCs to the market. Several strategies were proposed for large-area PSCs, such as blade coating [17–19], slot-die coating [20], screen printing [21] and spray deposition [22]. Among these methods, blade coating has the advantages of simplicity, high-speed and minimal solution loss, meaning that the blade coating technique is potentially suitable for scaling up production of PSCs.

Operationally, the blading process controls over solvent volatilization to tune film crystallization through the blade knife height, blading speed and blading temperature. Unfortunately, such control in blade coating is typically much less effective than that through antisolvent extraction in spin coating in combating the dendritic crystal growth, leading inevitably to the formation of numerous crevices in the resulting films. A possible chimie douse remedy for the blade coating is to rheologically, hydrodynamically and viscoelastically modify the perovskite precursor ink solution, termed pre-PVK ink hereafter, in the hope to balance the slow solvent volatilization and the dendritic crystal growth. Macropscopically, such pre-PVK ink is designed to possess an apt self-levelling property such that the resulting liquid film can uniformly cover the substrate. One the other hand, the microscopic crystallization process will also even out with such ink during the solidification process due to the space confinement imposed by the network structure mediated by some special self-assembling molecules such as surfactants.

In fact, gemini surfactant has already received attention in blade coating technology for perovskite solar modules. Deng et al. reported that small amounts of L-α- phosphatidylcholine (LP) surfactant could dramatically change the fluid drying dynamics and increase the adhesion of ink to the underlying non-wetting PTAA (poly(bis(4-phenyl) (2,4,6-trimethylphenyl) amine)) [18]. Some other surfactants have also shown positive effects on improving the quality of perovskite films [23, 24]. One of the observations about surfactant-assistance crystallization was that the zwitterionic LP is more effective than the non-ionic polyethylene glycol sorbitan monostearate (Tween 60), anionic sodium dodecyl sulfate (SDS) and cationic didodecyldimethylammonium bromide (DDAB) [18]. However, the mechanism of surfactant induced enhancement of perovskite film quality remains to be understood. LP is extracted from soybean or egg yolk with two alkyl chains of unequal lengths, and different product batches cannot guarantee a consistent molecular structure of LP [25]. In order to systematically study the influences of surfactant structure and length on the properties of pre-PVK ink during solidification, we successfully demonstrated the key role of a synthetic gemini surfactant with two equally long alkyl chains in assisting the blade coating of pre-PVK ink. The long alkyl chains are essential to lowering the critical micellar concentration (CMC) to the extent that a very small amount of surfactant additive will dramatically exert a positive influence on the rheological, hydrodynamic and viscoelastic properties of pre-PVK ink for blade-coating without compromising the transport properties perovskite films. Moreover, functionalized ionic moieties have strong capability to passivate the defects in the perovskite at the same time. Consequently, we were able to prepare high-quality 10 cm × 10 cm carbon-based modules with all of the functional layer blade coated in ambient air, and achieved a certificated efficiency of 15.46%. This significant step will promote the pace of real-world applications of all-printable perovskite photovoltaics.

## Experimental Section

### Materials

Unless stated otherwise, chemicals and solvents were obtained commercially and were used without further purification, including methylammonium iodide (MAI) and formamidine hydroiodide (FAI) (Dyesol, 98.0%), Lead iodide (PbI_2_) (TCI, 99.99%), SnO_2_ nanoparticle solution (15% in H_2_O colloidal dispersion, Alfa Aesar), graphene (Chengdu Organic Chemistry Co., Ltd., 98.0%). 1, 2-Distearoyl-sn-glycero-3-phosphocholine (DSPC, C_44_H_88_NO_8_P; DLPC, C_32_H_64_NO_8_P; DHPC, C_20_H_40_NO_8_P) (99.0%), Poly(3-hexylthiophene-2,5-diyl) (P3HT, 98%), N, N-dimethylformamide (DMF, 99.8%, anhydrous), Dimethyl sulfoxide (DMSO, 99.9%, anhydrous), Guanidine hydrochloride (CH_5_N_3_·HCl, 99%) and chlorobenzene (99.8%, anhydrous) were purchased from Sigma-Aldrich. Conductive carbon slurry was obtained from Guangzhou Saidi Technology Development Co., Ltd.

### Preparation of Devices

#### Preparation of Electron Transport Layer (ETL)

Laser-patterned ITO/glass substrates with a sheet resistance of 10 Ω sq^−1^ were precleaned using an ultrasonic washing with detergent, deionized water and ethanol for 10 min. SnO_2_ nanoparticle solution was dissolved with a volume ratio of 1:4 into deionized water, and small amount of CH_5_N_3_·HCl (4 mg mL^−1^) was added into the solution. The substrate was subjected with ultraviolet-ozone treatment for 20 min, and then SnO_2_ solution was bladed on the substrate with the size of 10 cm × 10 cm. The gap between substrate and D-bar was kept to be 100 μm, the coating speed was 300 cm min^−1^, and the substrate temperature of coater was kept to be 90 °C. After coating the SnO_2_ solution on the ITO substrate, the substrate was immediately transferred to the hot plate and annealed at 150 °C for 30 min. Before the preparation of ETL layer, ITO substrate was pre-patterned for P1.

#### Preparation of Perovskite Layer

Perovskite photoactive layer was deposited on SnO_2_ layer by blade-coating method. MA_0.65_FA_0.35_PbI_3_ precursor solution was prepared in a mixed solvent of DMF and DMSO (4:1, v/v) in nitrogen-filled glove box, and the concentration of solution was 1.2 M. According to different concentrations of DSPC in perovskite precursor solution (0, 0.04, 0.08, and 0.16 mg mL^−1^), we added corresponding DSPC mother solution into perovskite precursor solution. The gap between the coating blade and substrate was set as 200 μm. The coating speed was 300 cm min^−1^, and the substrate temperature of coater was kept to be 120 °C. After coating the perovskite precursor solution on the SnO_2_ layer, the substrate was immediately transferred to the hot plate and annealed at 150 °C for 10 min.

#### Preparation of Hole Transport Layer (HTL)

P3HT@Graphene as HTL was coated on perovskite layer. 20 mg P3HT and 10 mg graphene were dispersed in 2 mL chlorobenzene, and the mixture was stirred at 60 °C for 24 h for good uniformity. We then obtained the supernatant by centrifugation for 10 min. Before preparing the P3HT@Graphene film, ultrasonic dispersion was performed for 30 min. The gap between the coating blade and substrate was set as 100 μm. The coating speed was 300 cm min^−1^, and the substrate temperature of coater was kept to be 60 °C. After coating the P3HT@Graphene dispersion, the substrate was immediately transferred to the hot plate and annealed at 100 °C for 2 min. The P2 lines were patterned before the carbon coating process step.

#### Preparation of Carbon Electrode

The gap between the coating blade and substrate was set as 200 μm and the coating speed was 300 cm min^−1^. After coating the carbon electrode layer, the substrate was immediately transferred to the hot plate and annealed at 120 °C for 15 min, and then the P3 lines were patterned. It is worth noting that all functional layers were coated in the atmospheric environment.

## Result and Discussion

### Crystallization Kinetics Modulation with the Long-Chain Gemini Surfactant

**Fig. 1:**
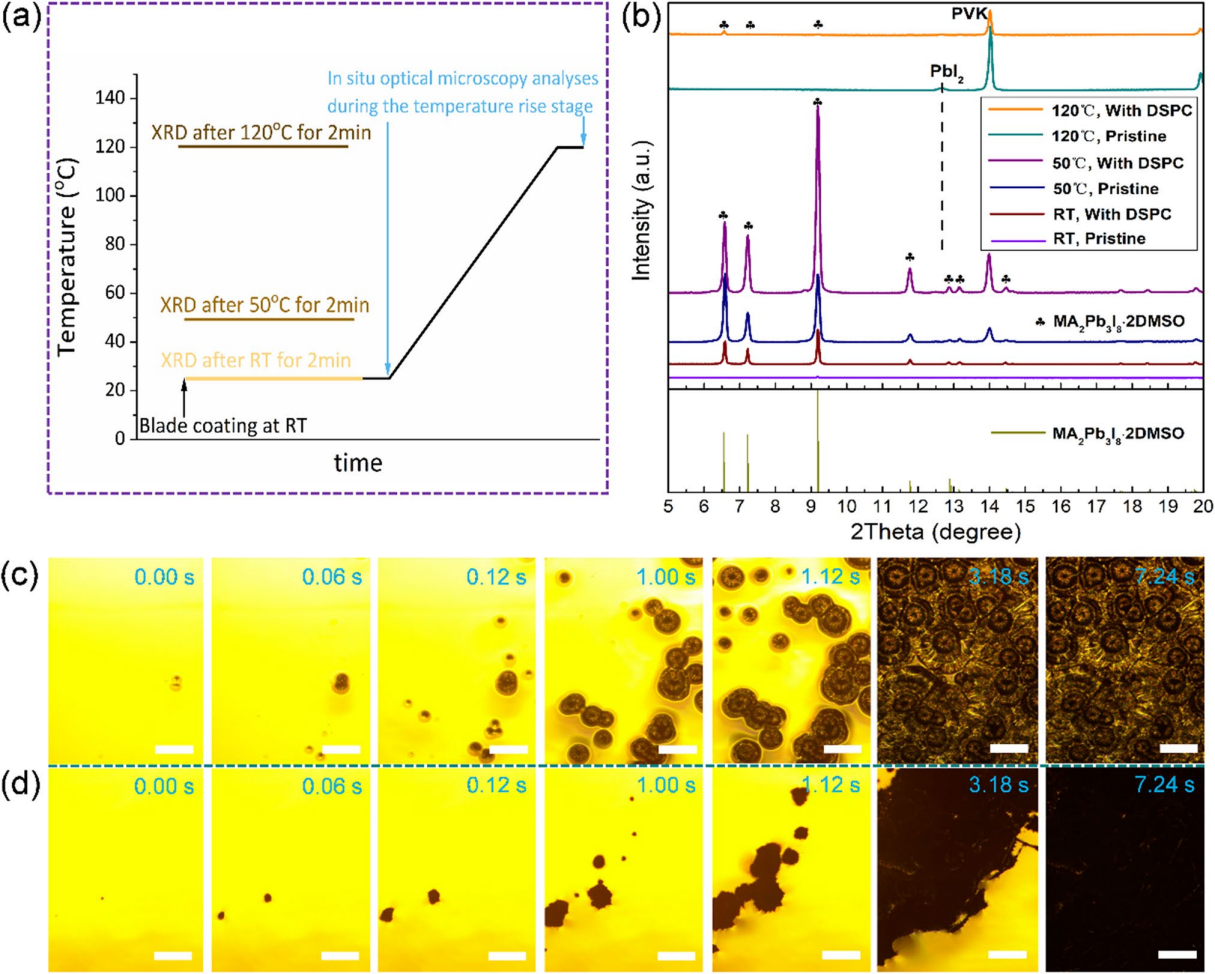
**a** Schematic diagram illustrating the sample conditions for the optical microscopic (Fig. 1c, d) and XRD (Fig. 1b).** b** XRD patterns of room temperature blade-coated pristine pre-PVK film and DSPC-pre-PVK film annealed at different temperatures for 2 min, in which clover symbol denotes the intermediate phase MA_2_Pb_3_I_8_·2DMSO. In situ optical microscopy images of **c** pristine and **d** DSPC pre-PVK ink annealing from room temperature to 120 °C, in which zero time is grabbed from 2.00 s in the Video S1 (The scale bar in all Figures is 100 μm)

To unlock the transformation process from the surfactant-containing pre-PVK ink film to the final perovskite film, the surfactant-assisted blade coating set-up was equipped with an optical microscope for direct observation. Let’s first look at the surfactant-free case. Here the liquid pre-PVK film was prepared by blade coating at room temperature and put on a hotplate, and the transformation of the pre-PVK film was observed in real time with the microscope during the annealing process (Fig. 1a). As shown in Fig. 1c and Video S1 (left), solid particles started to form in the liquid pre-PVK film immediately at 0.00 s. From 0.12 to 1.12 s, the solid particles grew up into radial spherules, concurrently with the appearance of more such solid particles covering a wider area of the view. From 1.12 to 3.18 s, the nucleation and growth of the particles continued and eventually formed different sizes of the radial spherules in contact with each other covering nearly the whole substrate surface but with obvious chinks and gaps between the neighboring spherules.

On close inspection, the radial spherules have grown into a multiple-ring pattern, possibly due to the growth in waves reminiscent of the coffee-ring structure. In general, the film is inhomogeneous and noncompact with the spherules stacked on each other. We believe that such radial spherules arise from self-assembly of one-dimensional intermediate crystals under the combined actions of concentration gradients and rheological and hydrodynamic properties of the unsolidified pre-PVK ink. In a previous work, we reported that the growth of one-dimensional (1D) intermediate phases were extremely fast in humid air [26]. When the solvent volatilized upon temperature rise, the concentration of pre-PVK ink increased and crystal nucleus of intermediate phases began to form. Indeed, the XRD patterns of pre-PVK ink film in Fig. 1b shows the characteristic peaks of the MA_2_Pb_3_I_8_·2DMSO intermediate phase as the temperature was increased [27, 28]. Note that the growth orientations of MA_2_Pb_3_I_8_·2DMSO crystals were not radial early on in the small emerging solid particles (nanoscale) simply because the precursor concentrations and rheological and hydrodynamic properties were relatively homogeneous in the bulk ink. However, when MA_2_Pb_3_I_8_·2DMSO grew into macroscopic solid particles (beyond micron) at 0.00 s as shown in Fig. 1c, the photographs show that the circumjacent liquid became more and more shadowy due to formation of the tiny solid particles spherically distributed around the central larger solid particles. Thereafter, the central solid particles grew radially, i.e., the 1D MA_2_Pb_3_I_8_·2DMSO fanned out, into the spreading shadows, resulting in more and more obvious bright rings with the increase of cluster size; for instance, at 0.12 s, the largest spherule exhibits the most obvious bright ring encircling it.

The appearance of the multiple-ring pattern after 1.00 s is phenomenologically similar to that from the well-known “coffee ring” effect in the pristine pre-PVK liquid, but the mechanistic details seem to be different. One can imagine, with the growth of 1D MA_2_Pb_3_I_8_·2DMSO, the solvent volatilization rate increased at the spherical edge, setting up a hydrodynamic, rheological and capillary field spherically distributed around the central solid particle. This view on the strong interplay between the central solid particle and the circumference is quite consistent with the growth trails we intercepted, such as radial clusters, multiple-ring pattern, shadowy circumference and bright ring. This inhomogeneous and uncontrollable growth mode heavily undermined the formation of high-quality perovskite films with the pristine pre-PVK liquid. The problem was the hierarchical formation of spherules starting from central solid particles surrounded by 1D arrays of smaller particles in the fluidic solvent. Eventually, upon solvent evaporation, the size-dispersed spherules are loosely stacked on each other forming a perovskite film with numerous wrinkles or cracks and with a poor coverage and coarse morphology, as shown in the photo at 3.18 s (Fig. 1c) and also in Fig. S1a.

To improve the perovskite crystallization process, we then introduced long-chain surfactants to modulate the rheological and hydrodynamic properties of the precursor solution. As shown in Fig. 1d and Video S1 (right), the transformation process in liquid DSPC-pre-PVK ink to solidified perovskite film was radically different from that in the pristine pre-PVK ink presented above. Although the solid particles were also observable at 0 s, all of those growth trails in pre-PVK ink film such as radial clusters, multiple-ring pattern, shadowy region and bright ring disappeared in the DSPC-pre-PVK ink film. Significantly, the growth process was much more smooth until the substrate was fully covered with a compact perovskite film at 7.24 s, which was much later than that in the pristine pre-PVK ink at 3.18 s. The reason behind it appears to be that adding a small amount of DSPC has dramatically changed the radial growth process of intermediate crystal spherules due to multiple changes in the liquid ink properties, including capillary, rheological, hydrodynamic, transport, nucleation, growth and transformation properties. As a result, liquid-mediated interference among the growing particles and radial spherule formation are suppressed by the surfactants, leading to more independent, uniform and smooth nucleation, growth and transformation of the particles in space and time. Figure 1b shows the XRD patterns highlighting the effects of DSPC. First of all, DSPC promoted the uniform nucleation of MA_2_Pb_3_I_8_·2DMSO intermediate phase even at room temperature, perhaps due to the strong interaction between the polar heads of DSPC with the colloidal particles, which will be further proved below by QCM-D analysis. Secondly, after annealing at 120 °C for 2 min, the peak of PbI_2_ appeared at 12.7° in the pristine pre-PVK ink but not in DSPC-pre-PVK ink. This indicates that the transformation from MA_2_Pb_3_I_8_·2DMSO to perovskite was much more controllable in the surfactant-containing precursor than in the pristine counterpart. Plausibly, adding DSPC promoted the uniform nucleation of MA_2_Pb_3_I_8_·2DMSO but decelerated the solidification to form perovskite, which can be seen from the lower perovskite peak intensity at 14.1° than in the pristine pre-PVK ink, in agreement with the above-presented in-situ optical microscope observations. In addition, the hydrophobic part of DSPC may have lessened the detrimental effect of water on the solidification process in humid atmosphere, which is known to cause uncontrollable transformation of the intermediate to PbI_2_ [26]. Figure S1b displays the final perovskite morphologies evolved from the DSPC-pre-PVK ink. Clearly seen are the large-size perovskite crystal grains with flat surfaces compactly joined together instead of the fissured spherical clusters arising from the “coffee ring”-like effect as in the perovskite film prepared with the pre-PVK ink.

To study the impacts of surfactant size on the properties of pre-PVK ink relevant to perovskite film formation, saturated phospholipids DHPC(20C), DLPC(32C), DMPC(36C), DPPC(40C), DSPC(44C), and DDOCPC(52C) with different alkyl chain lengths were selected. As shown in Fig. S2, these surfactants have the same polar head and the same two equal-length alkyl chain structure, but different alkyl chain lengths. The most obvious advantage of using these synthetic phospholipids for our study over natural phospholipids is that they have a definite molecular structure and high purity. As can be seen from Fig. S3, when the same mass of the gemini surfactant was added into DMF/DMSO, emulsification occurred in the solutions with DLPC and DSPC but not with DHPC, and moreover, DSPC showed most pronounced emulsification. This clearly demonstrates that the alkyl chain length of surfactant is critical to the lowering of critical micellar concentration (CMC) in DMF/DMSO [29]. The formation of micelles is expected to greatly change the rheological properties and viscoelasticity of the solution, which were then carefully investigated [30, 31].

**Fig. 2:**
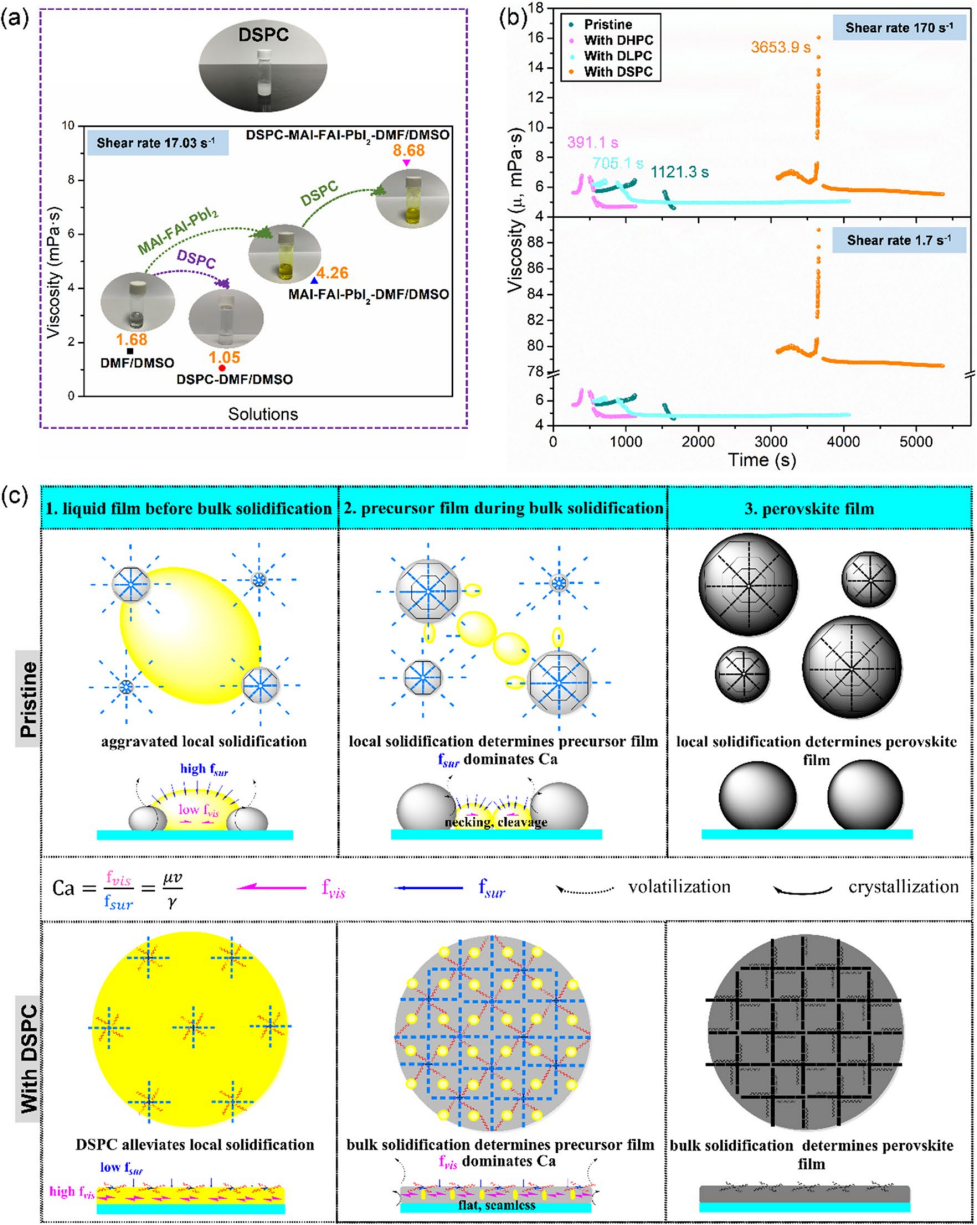
**a** Viscosities of the different solutions measured with a rotary viscosimeter at the shear rate 17.03 s^−1^. **b** Viscosity changes of the different precursor solutions with time under the shear rate of 170 and 1.70 s^−1^. **c** Schematic diagrams illustrating the blade coating processes with the pristine pre-PVK ink (top) and the DSPC pre-PVK ink (bottom). The yellow, gray and black regions depict the different solidification stages of liquid ink, solidified ink and perovskite film, respectively. Intermediate phases (INTs) between precursor and perovskite are denoted by blue rods. DSPC has two polar heads (denoted by two blue circles) with affinity interaction with INTs and two hydrophobic tails (denoted by red wavy lines) with repulsion interaction with INTs), and the unique interactions drive the arrangements of DSPC-INTs for the smooth growth to a compact and uniform perovskite film

Figure 2a exposes the influences of phospholipids on the viscosity of DMF/DMSO solutions. First, adding DSPC in DMF/DMSO actually decreased the viscosity from 1.68 to 1.05 mPa·s because the hydrophobic alkyl chains are dispersed in DMF/DMSO and as aprotic solvents, the DMF/DMSO cannot mediate strong interactions between micelles. However, the presence of PVK completely changed the picture; adding DSPC greatly increased the viscosity at the shear rate of 17.03 s-1 from 4.26 mPa·s (pre-PVK ink) to 8.68 mPa·s (DSPC-pre-PVK ink). Understandably, colloidal particles in the DSPC-pre-PVK ink have played an important role in increasing the viscosity by strongly interacting with the polar heads of DSPC in the DSPC-pre-PVK ink. Another important feature of the DSPC-pre-PVK ink is shear thinning, which is manifested in Fig. S4. Such a decrease of viscosity with shear rate is ascribable to the partial breakup of the self-assembled structures under shearing and the resulting reduction of attractive interactions between the loosen components. Shear thinning is a common phenomenon for many surfactant systems mainly caused by micelle shape changes under the shear force [32–34]. In our specific case, at a low shear rate, the colloidal particles and the micelles tend to be interlaced into a reticular structure, but increasing shear rate will weaken the linkages between the self-assembled species, thus decreasing the viscosity. Importantly, the viscosity of pre-PVK ink added with the short-chain gemini surfactants (SCGS) such as DHPC (20C) and DLPC (32C) failed to increase with and was actually independent of the shear rate. Plausibly, the SCGSs did not form micelles in the solvent system to begin with, so they failed to form the kind of reticular structure with the colloidal particles. Therefore, the use of long alkyl chains in surfactants is essential for increasing viscosity of the inks to control perovskite crystallization.

Next, we availed ourselves of quartz crystal microbalance with dissipation (QCM-D) to investigate the solidification process of pre-PVK ink. The differences of viscoelasticity between LCGS- and SCGS- pre-PVK inks were measured in real time to expose the influence of the alky chain length of surfactants on the solidification process. As shown in Fig. S5 for the solidification processes from 1 to 2, the solidification time of DHPC-pre-PVK ink (391.1 s) and DLPC-pre-PVK ink (705.1 s) is shorter than that of the pristine pre-PVK ink (1121.3 s), but the solidification of DSPC-pre-PVK ink (3653.9 s) takes much longer. It appears that the same polar heads of these three phospholipids play the role of accelerating solidification by promoting the intermediate phase formation. On the other hand, the increase of alkyl-chain length plays the role of slowing down solidification by increasing the viscosity and the capillary number of DSPC-pre-PVK ink. Understandably, both the increase of viscosity and capillary number (to be defined and discussed below) will degrade fluidity of the unsolidified liquid and limit the local inhomogeneous mass transport due to evaporation, flow and capillarity, thereby attenuating the interplay between the growing central particles and the circumjacent liquid.

Although the SCGS and LCGS are different in their ability to form the reticular structures needed for forming high-quality perovskite films, they both contribute to the favorable viscoelastic evolution during solidification, which is reflected by the changing trend of viscosity versus that of elastic modulus. To unravel it, we converted the data in Fig. S5 to the plots of Δviscosity relative to Δelastic modulus as shown in Fig. S6. The most important observation is that while for the processes from 1 to 2, the ascent rate of Δviscosity relative to Δelastic modulus is generally increased by the surfactants (DHPC, DLPC and DSPC), the descent rate of Δviscosity relative to Δelastic modulus for the processes from 3 to 4 (from unsolidified ink to residual solution) is generally reduced by the surfactants. In other words, by adding the surfactants to the ink, the relative viscosity increase of the solidifying film becomes faster, but the relative viscosity decrease of unsolidified ink becomes slower. Evidently, the solidification process with the surfactants has a higher impact on the viscosity of the solidifying film but a lower impact on the viscosity of unsolidified pre-PVK ink than that without the surfactants. Thus, the gemini surfactants, both SCGS and LCGS, can reduce the viscosity perturbation of unsolidified pre-PVK ink and increase the controllability of crystallization.

One of the film quality indicators is film roughness, which we will relate to the surfactant modulated solidification process of perovskite precursor inks. Figure S7 shows AFM images of the perovskite films fabricated using different surfactants along with roughness analysis. The most important observation is the opposite effect of SCGS on the film roughness to LCGS, and consequently the much higher roughness of the films fabricated with SCGS than that with LCGS. Specifically, with SCGS (DHPC, 22C), the perovskite film roughness increased from *R*_q_ = 109.0 to 131.0 nm, but with DLPC (32C) of an intermediate chain length and LCGS (DSPC, 44C), it decreased to 66.2 and 45.2 nm, respectively. As we have shown above, SCGS (DHPC, 22C) greatly shifts the start of solidification to an earlier time and reduces the controllability of crystallization, thus causing the roughness increase as observed. In contrat, LCGS (DSPC, 44C) greatly postpones the solidification start time and enhances the controllability of crystallization, thus causing the observed roughness decrease. Meanwhile, DLPC (32C) sits in between the two and has approximately the threshold alkyl chain length to impact on the desirable perovskite crystallization process. To sum up, both of the SCGS and LCGS can reduce the viscosity perturbation of unsolidified pre-PVK ink, but only when their alkyl chains reach a threshold length will the surfactants have a sufficient activity to greatly increase the viscosity and to postpone the solidification time. The surfactant activity of a gemini surfactant is directly reflected by the emulsification phenomenon, as clearly demonstrated in Figs. S3 and S8 for our solvent system. While the DHPC solution is still transparent at 2 mg mL^−1^, emulsification persists for DLPC even when the concentration is lower than 0.01 mg mL^−1^, meaning that the surfactant activity greatly increases with the alkyl chain length.

Since the SnO_2_ substrate for blade-coating is hydrophilic, especially after the plasma or ultra-violet ozone cleaning treatments, its interfacial tension with our ink liquids, even the pre-PVK ink liquid, is generally small, making the liquid self-levelling exceedingly well on the substrate during the blade-coating. As reported by Weinstein et al., a liquid coating on a substrate will continue to flow until immobilized by gelling, curing, dewetting or drying [35]. However, we need consider particle agglomeration caused by interface tension-driven flow. In our case, since the surface area per unit volume increases as the liquid ink is spread over a substrate, the volatilization of solvent speeds up at the gas–liquid interface near the contact line or the gas–liquid-colloidal particle boundary. This scenario is much more serious for the pristine pre-PVK ink film, giving rise to the interface tension-driven flow carrying the particles as shown in Fig. 2c. More specifically, the flow and evaporative perturbations during the blading process will induce local solidification and particle aggregation, which in return exacerbates the perturbations, and the vicious circle will result in a rough and inhomogeneous perovskite film. However, the DSPC surfactant can considerably alleviate the evaporative perturbations, liquid flow and particle aggregation by enhancing the viscous force over capillary force, thus permitting the formation of smooth, uniform and high-quality perovskite films. This is because the hydrophilic heads of gemini surfactant have an affinity interaction with the intermediate phase as well as the polar solvent, which drives the self-assembly of the surfactants into an ordered structure with an enhanced viscosity as shown in Fig. 2c. The real-time viscosity evolution in Fig. 2b demonstrates the pivotal role of the LCGS in retarding solidification of the liquid inks. Fundamentally, the surfactant activity of LCGS is superior to SCGS [30, 36, 37] in that only a very small amount is sufficient to reduce surface tension and optimize perovskite film morphologies without incurring significant adverse effects [38].

To quantify the surfactant effect on the blading process, capillary number (Ca) is perhaps a good metric, which is the dimensionless ratio of viscous force (f_*vis*_) to surface tension (f_*sur*_) [39–42]:

1 $$ {\text{Ca}} = \frac{{{\text{f}}_{{\rm vis}} }}{{{\text{f}}_{{\rm sur}} }} = \frac{\upmu \text v}{\upgamma } $$

where *μ* is the liquid viscosity, *v* is the liquid motion velocity, *γ* is the surface tension. Virtually, Ca can be used to distinguish two scenarios in our study: the interface tension-driven flow for pristine pre-PVK ink and the viscous stabilization for DSPC pre-PVK ink (Fig. 2c). In the pristine pre-PVK ink, Ca is dominated by high *f*_sur_ and low *f*_vis_, which makes the liquid ink susceptible to local flow and volatilization. Eventually, local solidification arising from uncontrolled perturbations will result in a low-quality perovskite film. In the DSPC pre-PVK ink, however, Ca is dominated by low *f*_sur_ and high f_*vis*_, which eliminates the influences of uncontrolled perturbations on the liquid film. Now the bulk solidification is completely driven by the uniform crystallization. Eventually, all of the liquid film, intermediate phase film, and perovskite film are flat and seamless owing to the assistance of DSPC in the blading process.

### Perovskite Films Blade-Coated with the Long-Chain Gemini Surfactant

**Fig. 3:**
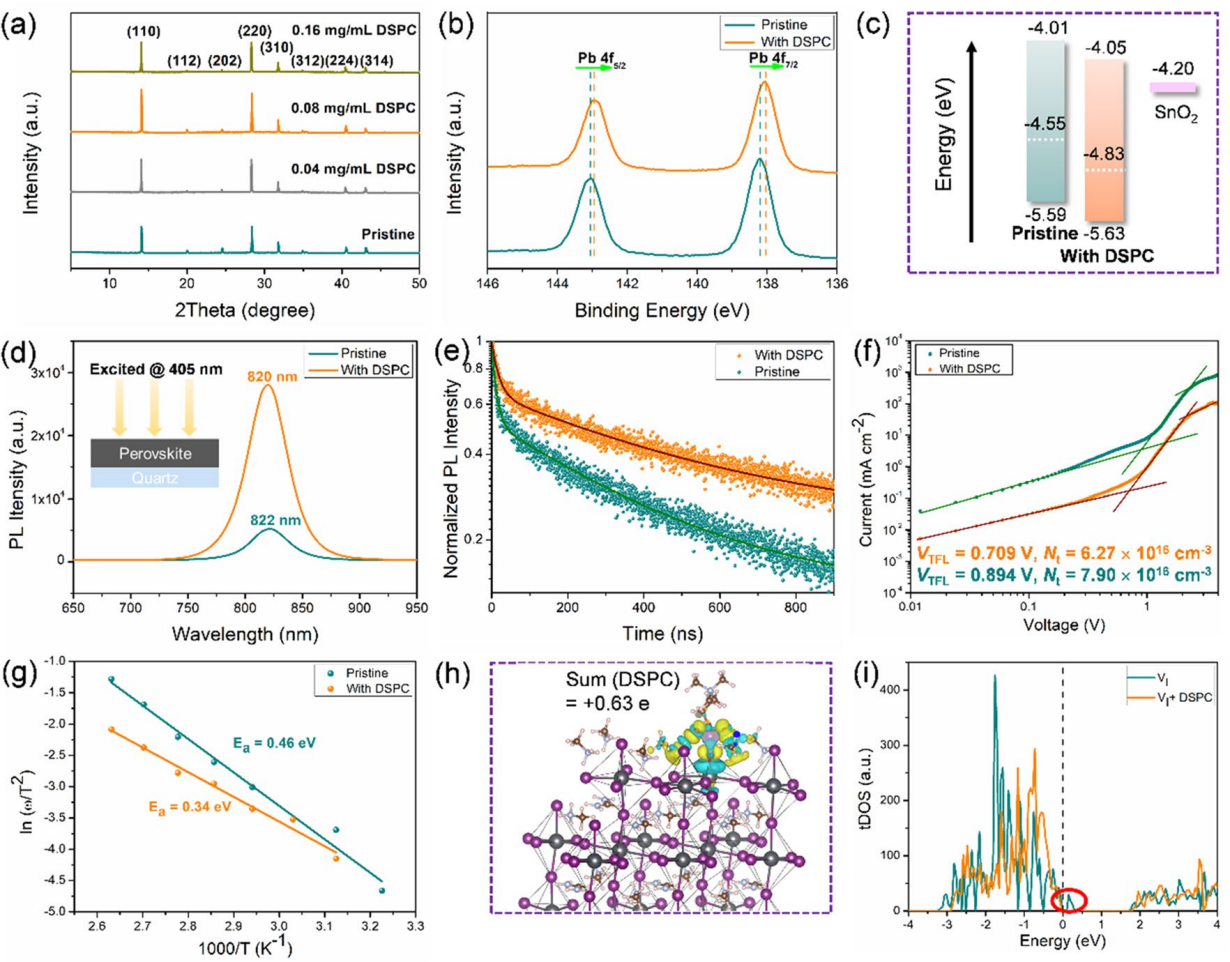
**a** XRD patterns of perovskite films with no and with different amounts of DSPC. **b** Pb 4*f* XPS spectra of the perovskite films with/without DSPC. **c** Energy band diagrams of the perovskite films with/without DSPC. **d** Steady-state PL spectra of the perovskite films with/without DSPC. All samples were prepared on quartz substrates and excited at 405 nm. **e** TRPL decays of the perovskite films with/without DSPC. **f** Space charge limited current (SCLC) curves for electron-only devices with/without DSPC. **g** Arrhenius plots of the characteristic transition frequencies of trap states in the devices with/without DSPC derived from thermal admittance spectroscopy. **h** Differential charge density distributions and Bader charge of perovskite interacting with DSPC. **i** tDOS of V_I_-containing perovskite with/without DSPC

In this Section, DSPC as the above-established LCGS is used to form perovskite films for the following study. The crystal structure of the annealed perovskite films obtained with different amount of DSPC doping was analyzed by XRD, and the result is shown in Fig. 3a. Generally observed are strong diffraction signals at 14.08°, 28.40° and 31.86°, which are assigned to (110), (220) and (310) planes of tetragonal perovskite, respectively. Minor diffraction signals at 19.98°, 24.48°, 34.95°, 40.65° and 43.17° are assigned to the (112), (202), (312), (224) and (314) planes, respectively [43]. As the DSPC content was increased from 0 to 0.16 mg mL^−1^, no diffraction peak shift was observed except for the highest diffraction peak intensities observed at the DSPC concentration of 0.08 mg mL^−1^. Obviously, DSPC did not permeate into the perovskite crystal lattice to induce a phase transition or form a certain 2D structure at perovskite surfaces and grain boundaries. As shown in Fig. S10, the annealing temperature was optimized at 150 °C for the highest crystallinity. In addition, the addition of DSPC almost did not change the UV–vis absorption spectrum and band gap of the perovskite (Fig. S11).

Steady-state photoluminescence (PL) and time-resolved photoluminescence (TRPL) spectroscopies were performed to study the electronic and optoelectronic qualities of perovskite films. As shown in Fig. 3d, compared with the pristine perovskite, PL intensity of DSPC-based perovskite was increased by a factor of 560%. In addition, there is a blue shift from pristine perovskite (822 nm) to DSPC-based perovskite (820 nm), suggestive of the suppressed non-radiative recombination by DSPC. TRPL profiles of the corresponding perovskite films are shown in Fig. 3e and the extracted parameters are summarized in Table S1. Two dynamic regimes were recognized: the fast decay lifetime τ_1_ is related to the charge trapping process at perovskite surfaces and interfaces, while the slow decay lifetime τ_2_ represents the de-trapping or recombination process in the bulk [44, 45]. The average fluorescence lifetimes of pristine and DSPC-based perovskite films are 189.52 and 292.05 ns, respectively, again evidencing the significantly less non-radiative recombination for the DSPC-based perovskite film. Additionally, adding DSPC significantly reduces the portion of τ_1_ (from A_1_ = 0.43 for pristine perovskite to A_1_ = 0.34 for DSPC-perovskite), further proving the effective suppression of non-radiative recombination in perovskite films, especially at perovskite surfaces and interfaces.

The perovskite films were carefully characterized to better understand the defect passivation effect of DSPC in the perovskite films induced by the interaction between DSPC and the defects. First of all, X-ray photoelectron spectroscopy (XPS) was used to investigate the coordination interaction involving DSPC in the perovskite films. As shown in Fig. S12, the pristine perovskite shows negligible O 1* s* signal, whereas the DSPC-perovskite presents an obvious O 1* s* signal at 532.40 eV, confirming the existence of DSPC in the perovskite film after thermal annealing. Moreover, as shown in Fig. 3b, the Pb 4*f*_7/2_ and Pb 4*f*_5/2_ peaks were shifted from 138.20 and 143.05 eV to low binding energies of 138.05 and 143.00 eV, presumably caused by donation of the lone pair electrons from the phosphate groups ((RO)_2_PO_2_^−^) of DSPC through the coordination interaction. Ultraviolet photoelectron spectroscopy (UPS) was used to investigate the energy band structure of perovskite. As shown in Fig. 3c, the Fermi level was determined to be − 4.85 and − 5.05 eV, respectively, for pristine and DSPC-based perovskite films, locating the corresponding valance band maximums (VBMs) at − 5.59 and − 5.63 eV. The narrowed energetic difference between Fermi level and VBM from 1.34 to 1.02 eV indicates the increased hole carrier concentration after the DSPC incorporation (Fig. S13).

The carrier mobility and defect density of perovskite film were estimated from space-charge-limited current (SCLC) measurement. The electron-only devices with the structure of ITO/SnO_2_/MA_0.65_FA_0.35_PbI_3_/PCBM/C were fabricated, and the device dark current was measured to derive the electron trap density. As shown in Fig. 3f, ohmic region, trap-filled limited (TFL) region and Child’s region could be clearly distinguished with respect to the applied potential. The introduction of DSPC shifted *V*_*TFL*_ from 0.894 to 0.709 V, hence reduced the trap density (*N*_t_) from 7.90 × 10^16^ to 6.27 × 10^16^ cm^−3^, demonstrating the passivation effect on the charge defects. Temperature dependent admittance spectroscopy (TAS) was carried out to analyze defects of the perovskite films. As shown in Fig. 3g, *E*_a_ of the trap energy level decreased from 0.46 to 0.34 eV after the DSPC incorporation, indicating the passivation of deep trap states. Additionally, the integrated trap density of the DSPC-based devices is 1.64 × 10^16^ cm^−3^, which is much lower than that of the pristine-based ones (2.26 × 10^16^ cm^−3^) (Fig. S14).

To further understand the interaction between DSPC molecule and perovskite surface, a theoretical model was constructed. For computational convenience, the defect passivation groups (= PO_4_^−^ and -NR_3_^+^) were used instead of the whole molecule, which is justified since there is no essential chemical difference between the two in terms of the defect passivation ability of the additive. The bonding structure between the additive and the perovskite crystal surface was obtained from the density functional theory (DFT) calculation. The charge difference and Bader charge analysis show that binding energy of DSPC at I vacancy is high (−5.76 eV), while it is low at A site vacancy (only −1.64 eV), i.e., the MA/FA sites. Charge density distributions of DSPC at I vacancy thereby are clearly shown in Fig. 3h, in which high local electron density is marked in yellow, and low electron density is marked in blue. It can be seen that there is a relatively high electron density around the oxygen atom with an average increase of 1.40 e due to the high electronegativity of oxygen, and there is a low electron density around the Pb atom with an average decrease of 1.07 e. Taking DSPC molecule as a whole for the calculation, there is an average gain of 0.63 e. This electron transfer from perovskite to DSPC molecule confirms the formation of chemical bond between the two. Furthermore, in order to investigate the effect of additives on the intragap states, the total density of states (tDOS) for the perovskite without and with DSPC was calculated, and the result is shown in Fig. 3i. Clearly, there are intragap states in the pristine perovskite associated with iodine vacancies (V_I_) in the perovskite (see the green line spectrum). To our delight, such intragap states could be eliminated by the DSPC molecule (see the yellow line spectrum), demonstrating its effectiveness for passivating the trap states. Since such intragap states in a semiconductor always serve as non-radiative recombination centers, their reduction will decrease the pernicious non-radiative recombination and improve photovoltaic performance of devices [46]. On the other hand, as shown in Fig. S15, the A sites hardly generated defect states. Figure S16 shows how passivation of the V_I_ defects in perovskite films could be achieved by LCGS through the O-Pb interaction.

### Photovoltaic Performance of the Fully Printed Modules

**Fig. 4:**
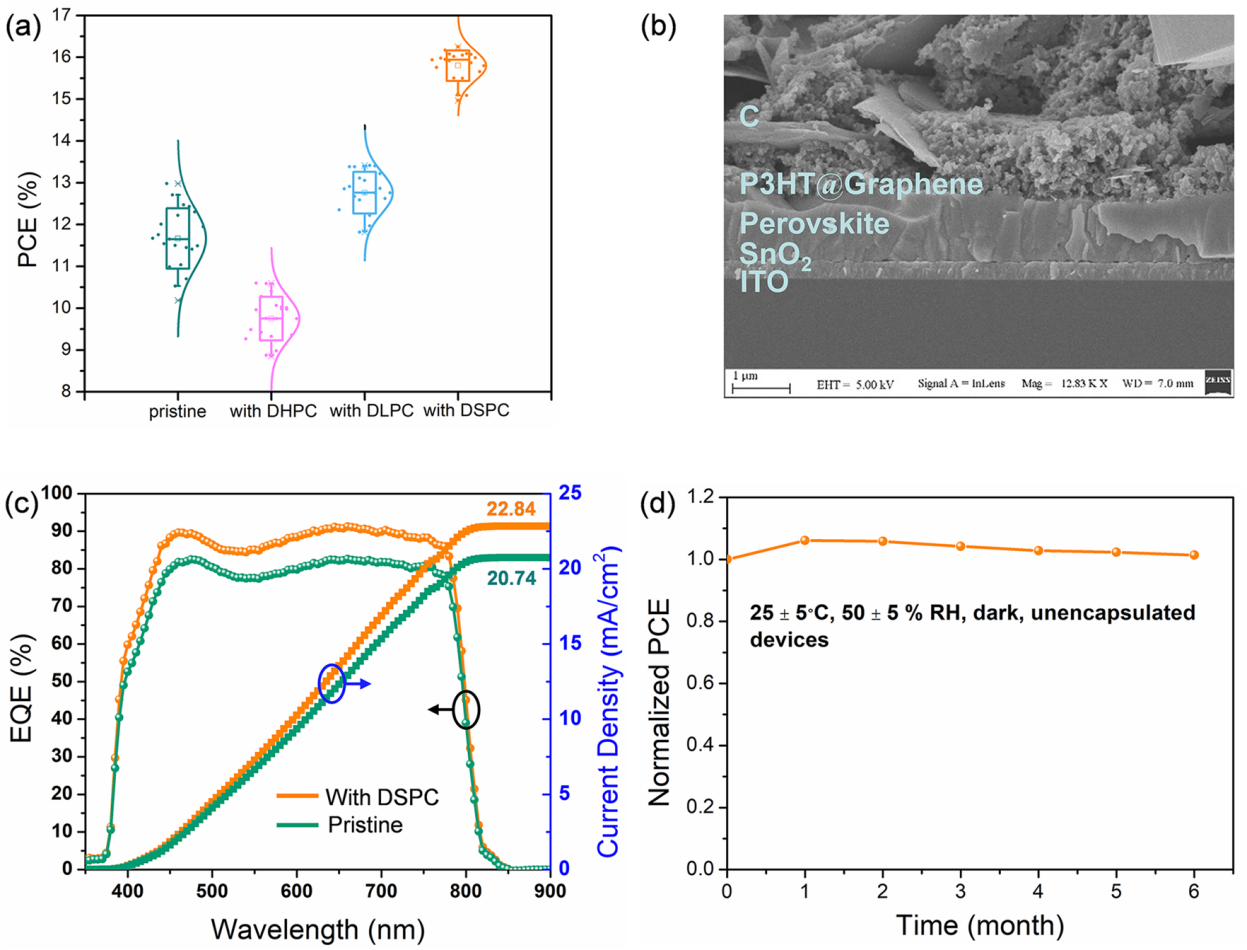
**a** Statistic distributions of PCE for the single sub-cell cut from the carbon electrode perovskite solar modules without or with different gemini surfactant doping (active area: 4.32 cm^2^). **b** SEM cross-section images of carbon electrode based perovskite solar modules. **c** IPCE spectra and integrated photocurrent curve of the devices with or without DSPC. **d** Long-term stability of the carbon electrode perovskite solar module with DSPC doping

We now turn to the photovoltaic performance of the perovskite modules prepared by the blade coating technique. The SEM images in Fig. 4b can be used to show the n-i-p module structure of ITO/SnO_2_/MA_0.65_FA_0.35_PbI_3_/P3HT@Graphene/C adopted in the present study. The perovskite solar module was fabricated on a 10 cm × 10 cm glass substrate, which provided an excellent demonstration of a printed large-area pin hole-free perovskite layer for high-efficiency solar modules^18^. The module was fabricated by etching the P1, P2 and P3 lines to connect ten sub-cells in series, as shown in Fig. S18. Figure 4a shows performance comparisons between the sub-cells in the modules based on the pristine perovskite and the perovskite with the different gemini surfactants with a mask hole set active area of 4.32 cm^2^. Remarkably, an average PCE of 15.80% was achieved for the sub-cells in the DSPC-perovskite based module, which is much higher than that of 11.67% for the pristine-perovskite based module. Figure S17 shows detailed comparisons of the measured photoelectric parameters including *J*_sc_, *V*_oc_, and *FF*. As expected, the better photovoltaic performance of the DSPC-perovskite based module is attributed to the much more homogeneous morphology and the longer carrier lifetime of the DSPC-based perovskite film with lower nonradiative recombination. Importantly, a certified efficiency of 15.46% with *V*_oc_ of 1.131 V and *J*_sc_ of 22.917 mA cm^−2^ was achieved for the fully blade coating prepared carbon based perovskite solar modules with a mask hole area of 4.32 cm^2^, as shown in Fig. S19. To the best of our knowledge, this represents the record efficiency, *V*_oc_ and *J*_sc_ for carbon electrode based perovskite solar modules (Table S2). The PV efficiency also increases with the length of alkyl chain. According to our latest laboratory results with another LCGS (DDOCPC, 52C), the PV efficiency has reached 17.05% for the module with HTL and 15.26% for the HTL-free module with an active area of 50 cm^2^ (Table S2 and Fig. S21).

The maximum power point (MPP) tracking profile is shown in Fig. S22 for the HTL-free PSC module under AM 1.5G illumination, and one sees a well stabilized power output in the operating state in ambient air. Clearly, the excellent defect passivation and the great improvement of perovskite film quality with the help of DSPC have effectively promoted the photovoltaic performance and the operational stability of perovskite solar modules. For a more life-like demonstration, Video S2 gives a flavor on the operation of the carbon-based perovskite solar modules under outdoor sunlight. The 30 cm × 30 cm array composed of 9 modules could easily power a household electric fan, further demonstrating the practicability and operation stability of carbon-based perovskite solar modules.

The *J*_sc_ is determined by the light absorbance and charge transport and collection efficiencies [47]. While the light absorption of the DSPC-based perovskite is slightly higher than that of the pristine one (Fig. S11c). In order to avoid the influence of the unilluminated sub-cell in the series module, single sub-cell was cut from the module to measure the external quantum efficiency (EQE) in Fig. 4c. The highest EQE is over 90% and the integrated current density is 22.8 mA cm^−2^, which indicates that the device with DSPC has a good light utilization due to the enhanced charge transport and collection efficiencies and due to the reduced recombination. Furthermore, we tested the long-term stability of the perovskite modules under a humid ambient atmosphere (25 ± 5 °C, 50 ± 5% RH). As shown in Fig. 4d, the devices were very stable, and to our surprise, the PCE actually increased by 1.4% after half a year. We suspect that the high module stability can be largely ascribed to the hydrophobic nature of carbon electrode which can prevent the perovskite layer from moisture penetration [48] and to the hydrophobic alkyl chains of DSPC molecules intertwined with perovskite which could create a barrier to moisture ingress and realize a sort of in-film encapsulation [49].

## Conclusions

Utility scale production of perovskite solar modules relies on low-cost printing of high-quality large-area perovskite films. However, we have shown that the pristine pre-PVK ink for blade coating suffers the interface tension-driven flow induced by local evaporation and solidification, leading to uncontrollable crystallization of intermediate phases and the eventual formation of holey and cracky perovskite films. To tackle this problem, we have demonstrated the critical roles of long alkyl chain gemini-surfactants in successfully blade coating uniform, dense and smooth large-area perovskite films. The rheological property and solidification process of the perovskite are greatly modified by the gemini surfactants with polar heads and hydrophobic tails, which mediate an orderly crossed assembly of the colloidal particles and thus greatly increase the viscosity of the pre-PVK ink. The large increase of viscosity pushes up the viscous force against the capillary force, i.e., the capillary number, according to the lubrication approximation theory. The key is a large enhancement of capillary number in the perovskite ink with only a tiny amount of DSPC, which retards the fluidity, decreases the volatilization rate, extends the solidification time, and thus considerably alleviates the influences of uncontrollable solidification during the blade coating process. Consequently, the coverage and smoothness of perovskite films are significantly improved, allowing to achieve high-efficiency perovskite solar modules. The surfactant additive, albeit in a very small amount to not compromise the optoelectronic properties of perovskite, could effectively modulate perovskite crystallization kinetics, optimized energy level alignment, and neutralized charge defects. A record certificated efficiency of 15.46% for the fully blade-coated carbon-based perovskite solar modules was achieved with the assistance of DSPC doping, and the efficiency even increased by 1.4% after 6 months of storage in a humid ambient atmosphere. This work paves the way for pushing the fully printed and highly-efficient and stable perovskite solar modules towards commercialization.

### Supplementary Information

Below is the link to the electronic supplementary material.

Supplementary file1 (PDF 2028 kb)

Supplementary file2 (MP4 8264 kb)

Supplementary file3 (MP4 20508 kb)
