# Supplementary material for: Long-Chain Gemini Surfactant-Assisted Blade Coating Enables Large-Area Carbon-Based Perovskite Solar Modules with Record Performance
Source: Nanomicro Lett. 2023 Jul 14;15:182. doi: 10.1007/s40820-023-01155-w (PMC10349030; doi:10.1007/s40820-023-01155-w)
Supplement: Supplementary file 1 — Supplementary file1 (PDF 2028 kb) [file 40820_2023_1155_MOESM1_ESM.pdf]

Supporting Information for

Long-Chain Gemini Surfactant-Assisted Blade Coating Enables Large-Area Carbon-Based Perovskite Solar Modules with Record Performance

Yumin Ren[1, †], Kai Zhang[1, 2, †], Zedong Lin[1, 2], Xiaozhen Wei[1, 3], Man Xu[1], Xianzhen Huang[1], Haining Chen[3] and Shihe Yang[1, 2, *]

[1] Guangdong Provincial Key Lab of Nano-Micro Material Research, School of Chemical Biology and Biotechnology, Shenzhen Graduate School, Peking University, Shenzhen, 518055, P. R. China

[2] Institute of Biomedical Engineering, Shenzhen Bay Laboratory, Shenzhen, Guangdong, 518107, P. R. China

[3] School of Materials Science and Engineering, Beihang University, Beijing, 100191, P. R. China

[†] Yumin Ren and Kai Zhang contributed equally to this work.

*Corresponding author. E-mail: chsyang@pku.edu.cn (Shihe Yang)

S1 Supplementary Experimental Section

S1.1 Density Functional Theory (DFT) Calculation

DFT simulation is performed by running the Vienna ab initio simulation package (VASP)[S1]. Projector-augmented wave (PAW) is used to describe the core-valence electron interaction[S2]. Generalized gradient approximation (GGA) in Perdew, Burke, and Ernzerhof (PBE) is adopted to treat the exchange correlation functional[S3]. The energy cutoff for wave function expansion is set as 400 eV. For structure relaxation, the energy convergence criterion is set as 1×10^{-5} eV. The ion force convergence criterion is set as 0.03 eV/Å. The G-centered k-point mesh is set as $3 \times 2 \times 1$.

The bulk $MAPbI_3$ with the space group PNMA (a = 8.56 Å, b = 9.25 Å, c = 12.96 Å, $\alpha = \beta = \gamma = 90^\circ$) is cleaved along the (0 0 -1) plane with I as termination. A 20 Å vacuum layer was added to separate the interaction between periodic images. Then, we expand it as $1 \times 2 \times 1$ supercell ($MAPbI_3$ (0 0 -1)). The 1/3 of MA molecules in $MAPbI_3$ (0 0 -1) is replaced by the FA molecules to construct the $MA_{0.65}FA_{0.35}PbI_3$ (0 0 -1). For investigation of I vacancy defect, we delete a I atom on the termination of $MA_{0.65}FA_{0.35}PbI_3$ (0 0 -1) to construct a I vacancy V_I. The DSPC molecule is too large for DFT calculation, so we choose the group in DSPC molecule which has the defect passivation function for calculation. The defect passivation group of DSPC molecule is added on the V_I site of $MA_{0.65}FA_{0.35}PbI_3$ (0 0 -1) to construct V_I-DSPC. For investigation of A site vacancy defect, we delete a MA atom on the termination of $MA_{0.65}FA_{0.35}PbI_3$ (0 0 -1) to construct the A site vacancy V_A. The defect passivation group of DSPC molecule is added on the V_A site of $MA_{0.65}FA_{0.35}PbI_3$ (0 0 -1) to construct V_A-DSPC.

S1.2 Characterization

The morphology of perovskite films was characterized by field emission scanning electron microscopy (JEOL 7100F) equipped with an electrically cooled X-ray

spectrometer. Crystal structure identification was determined by X-ray diffraction (XRD, Philips PW-1830 X-ray diffractometer, λ = 1.5419 Å, 298 K, Cu Kαradiation). Viscosity of solutions was measured by viscometer (TOKI SANGYO TV-25). Steady-state photoluminescence (PL) spectrograms were measured on fluorospectrophotometer (Horiba, FluoroMax-4) with excitation wavelength of 405 nm. Time-resolved photoluminescence (TRPL) was determined by the time-correlated, single-photon counting technique (pico1000, Dalian Institute of Chemical Physics, China). UV-vis absorption spectrograms were measured on a UV-vis spectrometer (UV-1800, Shimadzu). AFM images were collected on a Bruker Dimension Icon using Peak Force Tapping Mode in air.

Chemical state and qualitative analysis of perovskite surface elements were measured by X-ray photoelectron spectroscopy (XPS, Thermo Fisher ESCALAB 250Xi). Temperature dependent admittance spectroscopy (TAS) was measured on Zahner electrochemical workstation in the dark at various temperatures (T = 280-380 K) controlled by a liquid nitrogen cryostat (Oxford Instruments, OptistatDN). External quantum efficiencies (EQE) were measured by a Zolix detector responsivity measurement system calibrated by a standard silicon cell. Space-charge limited current (SCLC) data were collected by measuring the dark I-V curve on a Zahner Zennium electrochemical workstation. Current-voltage (J-V) characteristics of devices were recorded by a Keithley 2400 source meter under 1 Sun (AM 1.5 G, 100 mW cm^{-2}, the light intensity was calibrated using a certified standard silicon cell) illumination with a solar simulator (Newport, Oriel Sol3A).

S1.3 Analysis of In-situ Optical Microscope

The in-situ optical microscope images were observed by a polarizing microscope (Shanghai Bimu Instrument, XPY-800E), and the automatic exposure high-speed camera (TOUPCAM, UCMOS05100KPA) was used to capture the images and shoot videos of the whole process. The optical microscope videos were shot under the flowing conditions. The liquid pre-PVK film was bladed at room temperature, and then put on a hotplate at room temperature. The temperature was set as room temperature and slowly raised to 120 °C at a constant rate. The transformation from liquid film to perovskite film was slower than the direct annealing at 120 °C, which was easy to record the transformation process.

S1.4 Analysis of Quartz Crystal Microbalance with Dissipation (QCM-D)

QCM-D can be used to characterize soft materials at the molecular level, in which dissipation (damping) is determined by the stop time of the oscillation after the power disconnecting. Structural properties of film are measured by changes of dissipation at different frequency multiplications. By tracking changes of frequency and dissipation, the viscoelastic information of soft film can be obtained, which is suitable for a wide range of rheological and phase transition applications. Processes such as crosslinking, swelling, entanglement, aggregation, and other conformational changes can be tracked in real time by monitoring viscoelastic changes. In this work, QCM-D data of pre-PVK ink film was measured and fitted respectively by the instrument of QSense Explorer and the software of QSense Dfind (Biolin Scientific Co., Ltd.). 1uL pre-PVK ink spread on the gold electrode as the testing film of pre-PVK ink, then the electrode was placed

into the testing chamber. 500 uL/min air blow into the chamber to promote the volatilization of solvent and solidification of the pre-PVK ink film. Thus the raw data that the frequency and dissipation changed with time at different frequency multiplication were acquired. The raw data were fitted by the Broadfit model of QSense Dfind. Therefore, the changes of viscoelasticity were obtained and plotted in Figure S5, in which Δviscosity and Δelastic modulus denoted the relative values between the detectable viscoelasticity in the real-time liquid and the reference viscoelasticity in the initial bulk liquid. The changes of viscosity reflected changes of the proportion of colloidal solids and the degree of interlacement. The changes of elastic modulus reflected the degree of solidification. When bulk liquid began to transform into rigid film through the volatilization of solvent and the crystallization of intermediate phases, the values of Δviscosity and Δelastic modulus had a sudden increase (the steep curve from 1 to 2 and the time of the highest point shown in Figure S5). When the bulk film approached the state of rigid film, the frequency of bulk film was out of the detection range, so the record of solidification process discontinued and the instrument turned to record the unsolidified ink which had similar testing frequency to the reference ink. The solutes of unsolidified ink were further consumed during the process of aging or growth until the solvent was completely removed (the decline curve from 3 to 4 in Figure S5). Due to the negative Δelastic modulus cannot be calculated by the fitting software, the decline trends were symbolized by the arrows below 0 in Figure S5 and Figure S6.

S1.5 Analysis of Time-Resolved Photoluminescence (TRPL)

The TRPL decay curves were fitted to a biexponential rate law:

$$(t) = A_1 \exp\left(-\frac{t}{\tau_1}\right) + A_2 \exp\left(-\frac{t}{\tau_2}\right) \qquad \text{(S1)}$$

where A_1 and A_2 are the relative amplitudes, and $\boldsymbol{\tau}_1$ and $\boldsymbol{\tau}_2$ are the lifetimes for the fast and slow decays, respectively.

S1.6 Analysis of Space-Charge-Limited Current (SCLC)

The trap-filled limit voltage was determined by the trap density (N_t):

$$V_{TFL} = \frac{eN_tL^2}{2\varepsilon_0\varepsilon_r} \qquad \text{(S2)}$$

where V_{TFL} is the trap filled limit voltage, e is the elementary charge, L is the thickness of perovskite, $\boldsymbol{\varepsilon}_r$ is the relative dielectric constant of perovskite, and $\boldsymbol{\varepsilon}_0$ is the vacuum permittivity.

S1.7 Analysis of Temperature Dependent Admittance Spectroscopy (TAS)

The capacitance spectra was measured from 280 to 380 K in 10 K increments in the dark from 10^2 to 10^5 Hz. An AC voltage of 5 mV was used as an excitation signal and DC bias was kept at 0 V during the measurement. The trap energy level (E_a) was determined by the equation.

$$\omega_0 = \beta T^2 \exp\left(\frac{-E_a}{K_bT}\right) \qquad \text{(S3)}$$

where ω_0 is the characteristic transition frequency, β is a temperature-independent

parameter, K_b is the Boltzmann's constant, and T is the temperature, respectively. The trap density (N_t) can be derived according to the following equation.

$$N_t(E_\omega) = -\frac{V_{\mathrm{bi}}}{eW}\frac{dC}{d\omega}\frac{\omega}{K_bT} \quad \text{(S4)}$$

where V_{bi} is the built-in potential, e is the elementary charge, W is the depletion width, C is the capacitance, ω is the applied frequency, respectively. The values of V_{bi} and W could be obtained from the Mott-Schottky analysis. The demarcation energy (E_ω) was obtained from the equation.

$$E_\omega = K_bT\ln\frac{2\beta_cN}{\omega} \quad \text{(S5)}$$

where β_c is the capture coefficient for the charge carriers, and N is the effective density of states in a given band in the devices, respectively.

Table S1

Sample	A_1	τ_1 (ns)	A_2	τ_2 (ns)	Average decay time[a] τ (ns)
Pristine	0.43	11.65	0.39	385.63	189.52
DSPC-based	0.34	17.67	0.43	509.01	292.05

[a]Average decay time was calculated according to the equation: $\tau = (A_1\tau_1+A_2\tau_2)/(A_1+A_2)$.

Table S2 The reported efficiencies of carbon-based perovskite solar modules

Reference	V_{oc} (V)	J_{sc} (mA/cm^2)	*FF*	Active Area (cm^2)	PCE (%)
[S4] HTL-free<br>10 sub-cell module	9.3	2.0	0.56	49	10.4
[S5] HTL-free<br>4 sub-cell module	3.77	5.24	0.47	31	9.11<br>(certified)
[S6] HTL-free<br>8 sub-cell module	7.05	2.25	0.70	47.6	11.16
[S7] HTL-free<br>9 sub-cell module	8.50	2.50	0.61	60.08	12.87
[S8] Single sub-cell from module	1.05	21.2	0.69	4	15.3
This work (C44)<br>Full blade coating<br>Single sub-cell from module	1.131	22.917	0.60	4.32	15.46 (certified)
This work (C52, the latest)<br>Full blade coating<br>10 sub-cell module	10.75	2.37	0.67	50.00	17.05
This work (C52, the latest)<br>Full blade coating<br>HTL-free<br>10 sub-cell module	10.46	2.45	0.60	50.00	15.26

S2 Supplementary Figures

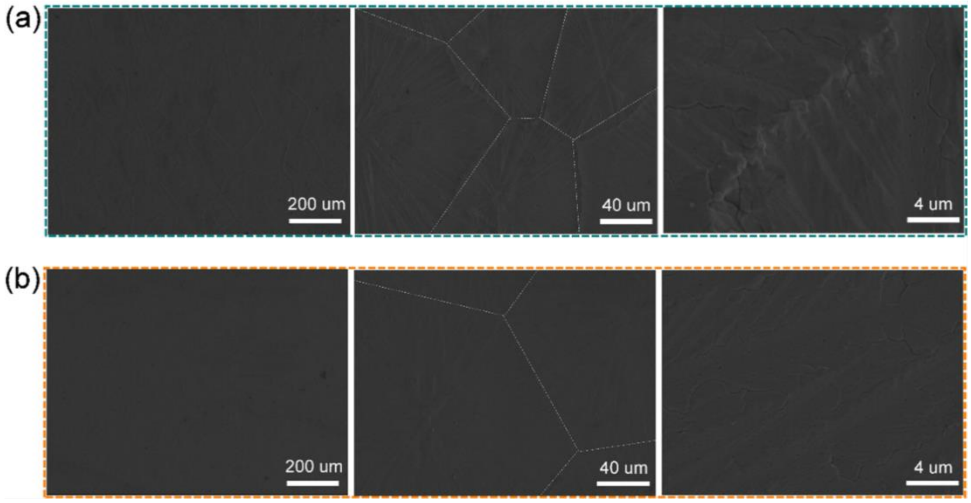

Fig. S1 SEM images of the perovskite films prepared **a)** without DSPC, and **b)** with DSPC

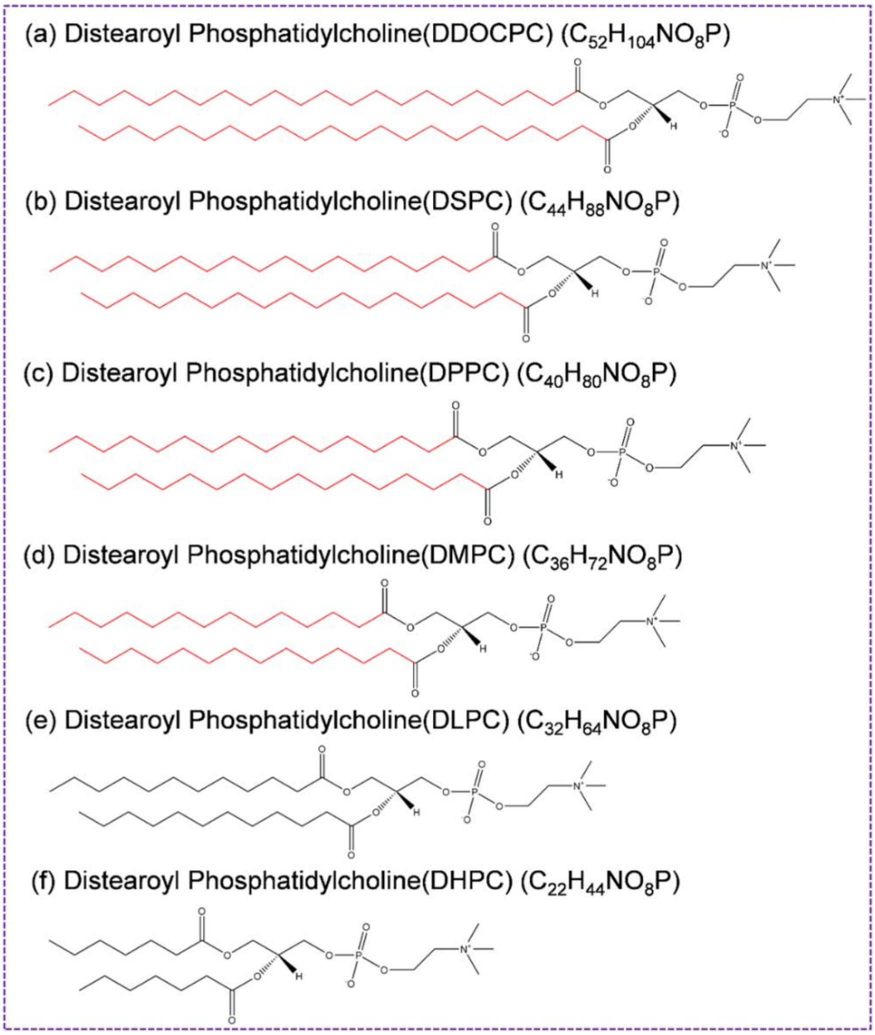

Fig. S2 Molecular structure of **a)** DDOCPC, **b)** DSPC, **c)** DPPC, **d)** DMPC, **e)** DLPC, and **f)** DHPC

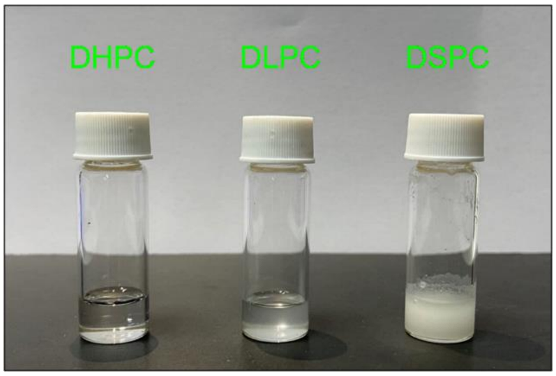

Fig. S3 Photos of 1mg DHPC, DLPC and DSPC in 1mL solvent of DMF/DMSO (4:1, v/v)

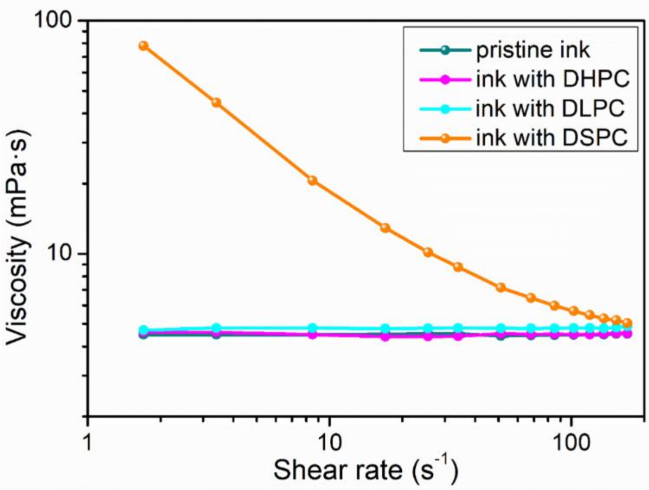

Fig. S4 Relationship between viscosity and shear rate for different liquid inks

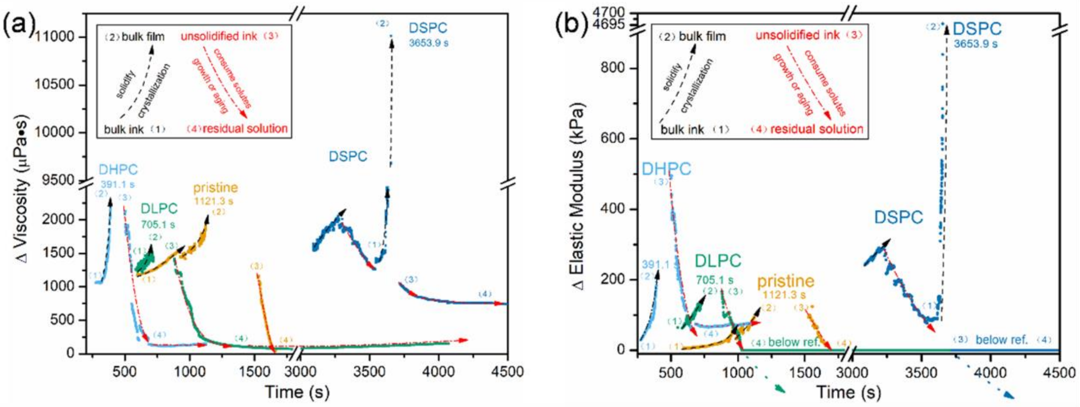

Fig. S5 The difference values of **a)** viscosity and **b)** elastic modulus relative to the reference values of bulk inks. The black arrows (the process from 1 to 2) denote the changes from liquid bulk ink to solid bulk film, and the red arrows (the process from 3 to 4) denote the changes from unsolidified ink to residual solution. The time values are taken from the highest point of 1 to 2 that is detectable by QCM-D. Since the curves below the reference values cannot be obtained by calculation, they are taken to have 0 value, for example, the elastic modulus curves from 3 to 4 in DLPC- and DSPC-pre-PVK ink. According to the results of XRD in **Fig.** 1c and QCM-D in **Fig.** S5, the formation of $MA_2Pb_3I_8$•2DMSO is promoted by the polar head of DSPC

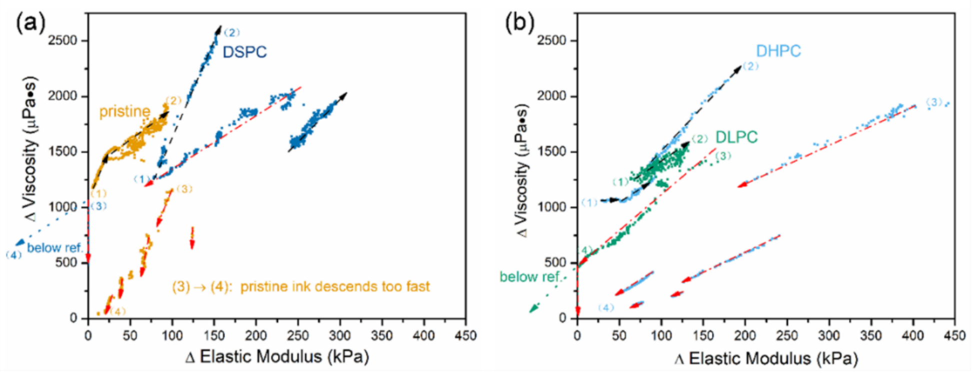

Fig. S6 The relationship between viscosity increment and elastic modulus increment in **a)** the pristine and DSPC-pre-PVK inks; **b)** the DHPC- and DLPC-pre-PVK ink. The difference values of viscosity and elastic modulus relative to the reference values of bulk inks are converted from **Fig. S5** (the relationship between change and time). Likewise, the black arrows (the process from 1 to 2) denote the changes from bulk ink to bulk film, the red arrows (the process from 3 to 4) denote the changes from unsolidified ink to residual solution. Since the curves below the reference values cannot be obtained by calculation, they are taken to have 0 value, for example, the elastic modulus curves from 3 to 4 in DSPC- (a) and DLPC-pre-PVK (b) inks

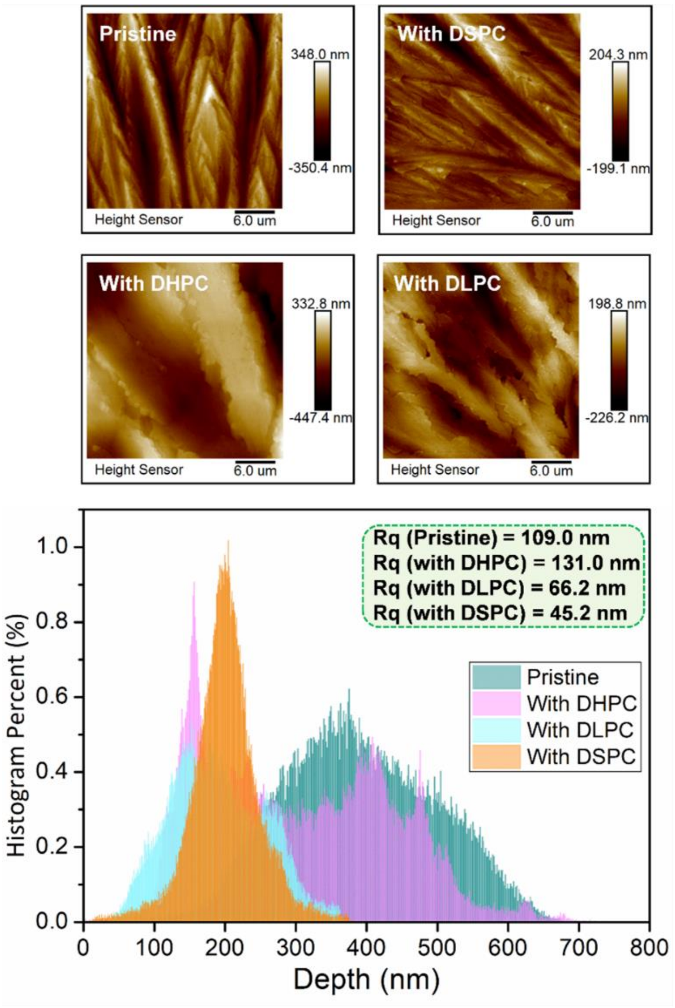

Fig. S7 AFM images (above) and depth histogram statistics (bottom) of the perovskite films prepared with/without the gemini surfactants

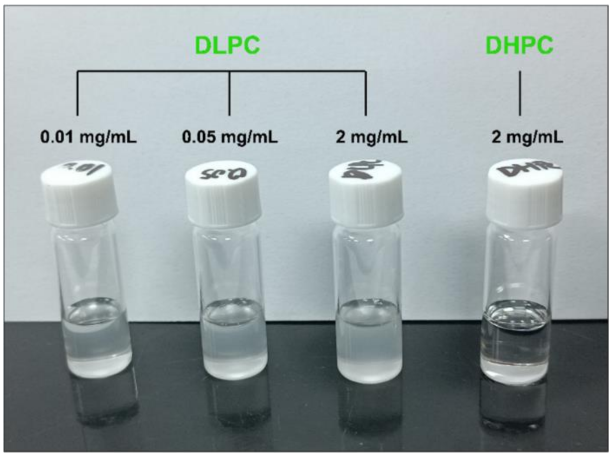

Fig. S8 Photos of 0.01mg DLPC, 0.05 mg DLPC, 2 mg DLPC and 2 mg DHPC in 1 mL solvent of DMF/DMSO (4:1, v/v)

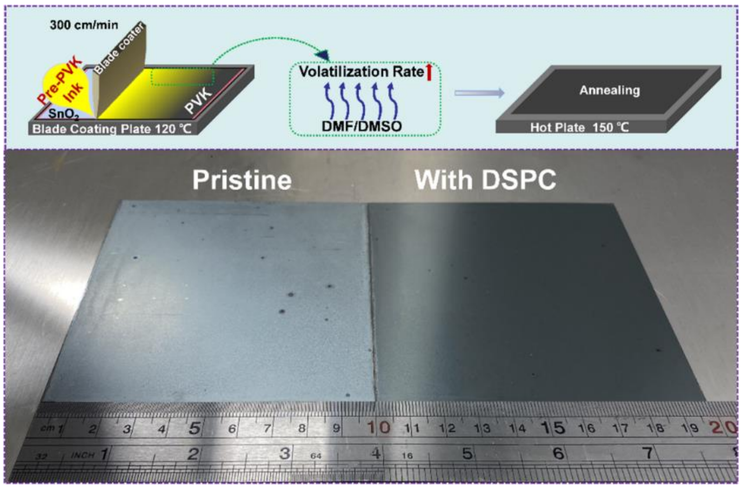

Fig. S9 Top: schematic of the blade-coating process; Bottom: Photos of the blading-coated perovskite films. Bottom left: without DSPC; Bottom right: with DSPC

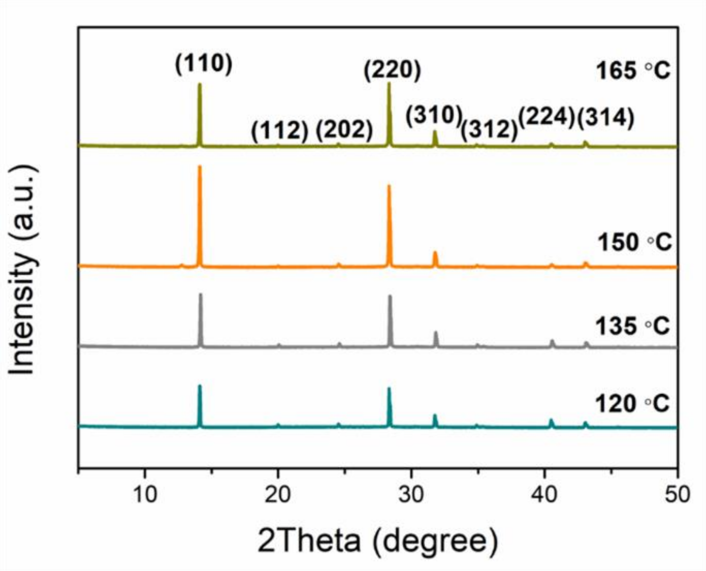

Fig. S10 XRD patterns of perovskite films with DSPC at different annealing temperatures

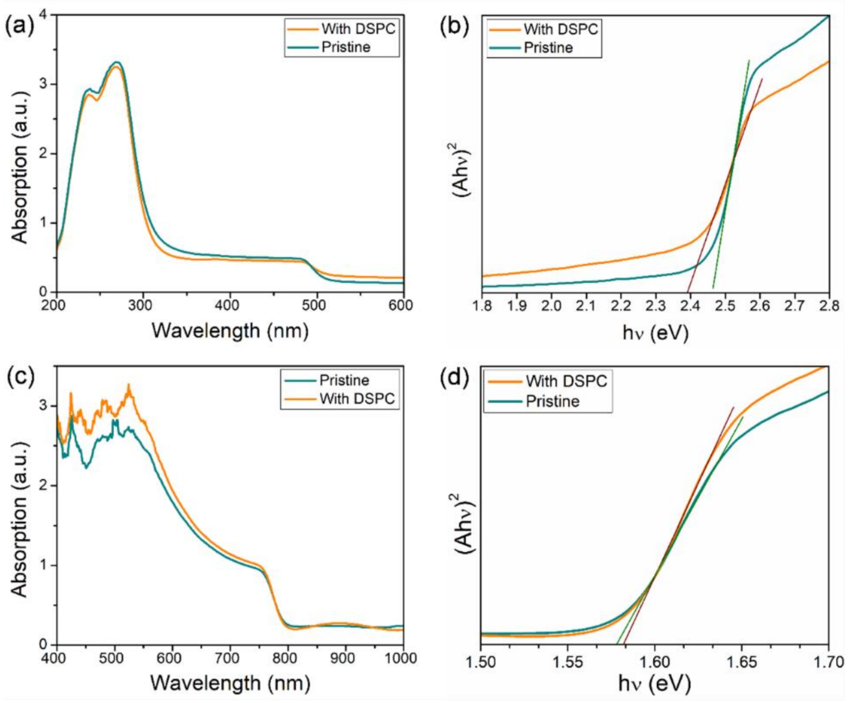

Fig. S11 UV-vis absorption spectra of **a)** perovskite precursor solutions and **c)** perovskite films; Tauc plots as a function of $(Ah\nu)^2$ versus energy for **b)** perovskite precursor solutions and **d)** perovskite films

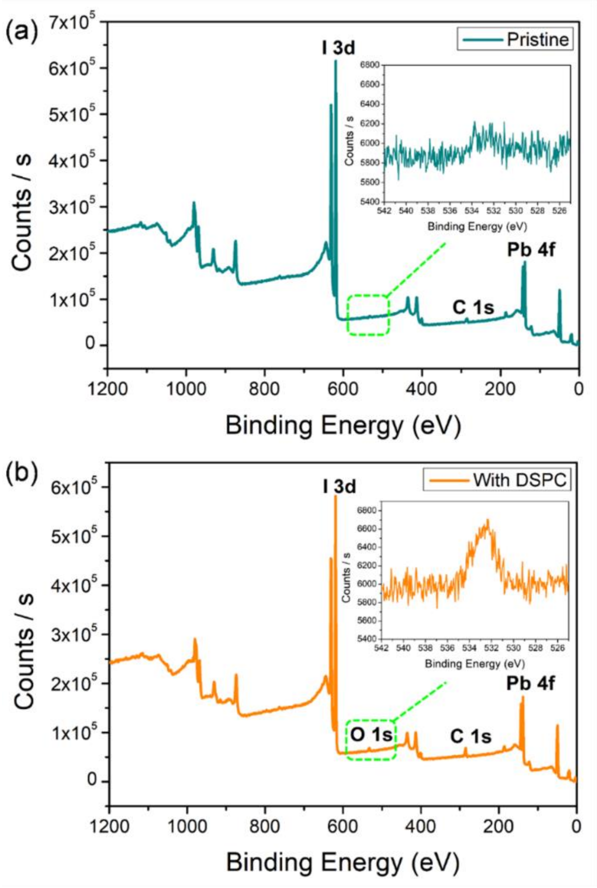

Fig. S12 XPS survey spectra and the O 1s spectra (insets) of perovskite films. Above: without DSPC; Below: with DSPC

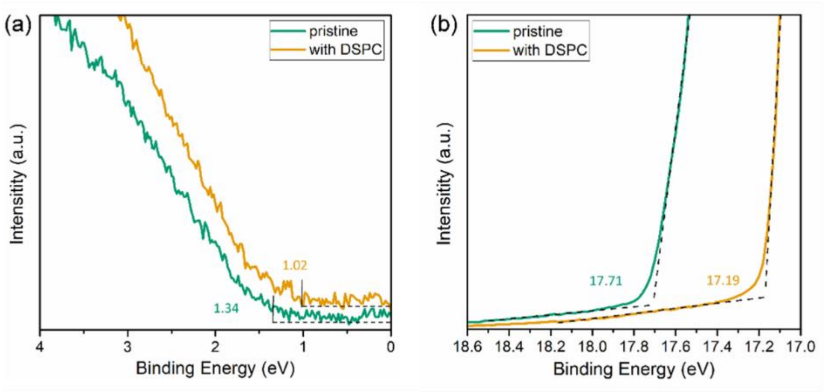

Fig. S13 UPS analyses of perovskite films prepared with/without DSPC

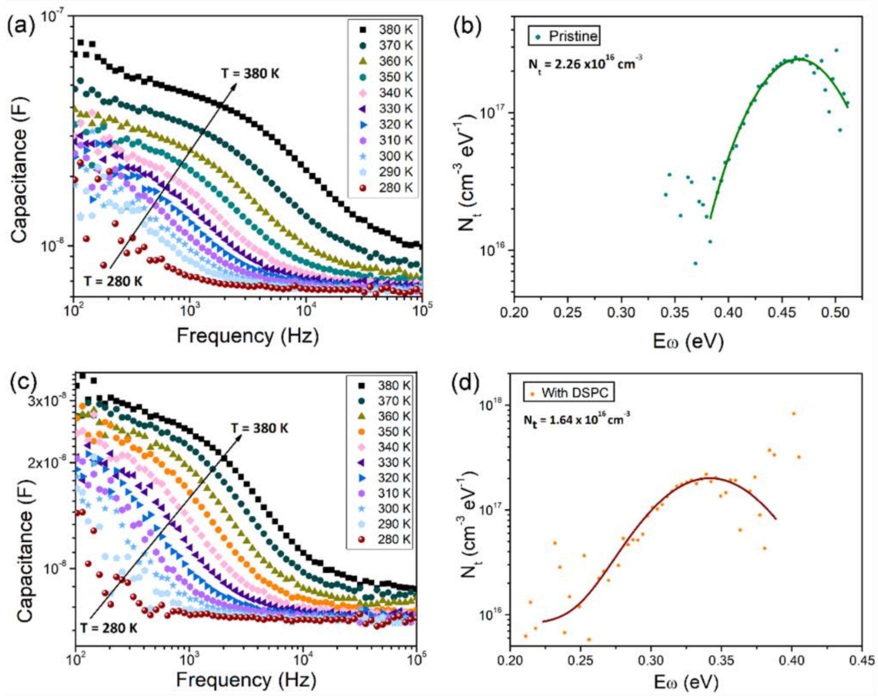

Fig. S14 Temperature dependent capacitance spectra of perovskite **a)** without DSPC and **c)** with DSPC. Density of trap states in perovskite obtained from the thermal admittance spectroscopy measurements **b)** without DSPC and, **d)** with DSPC

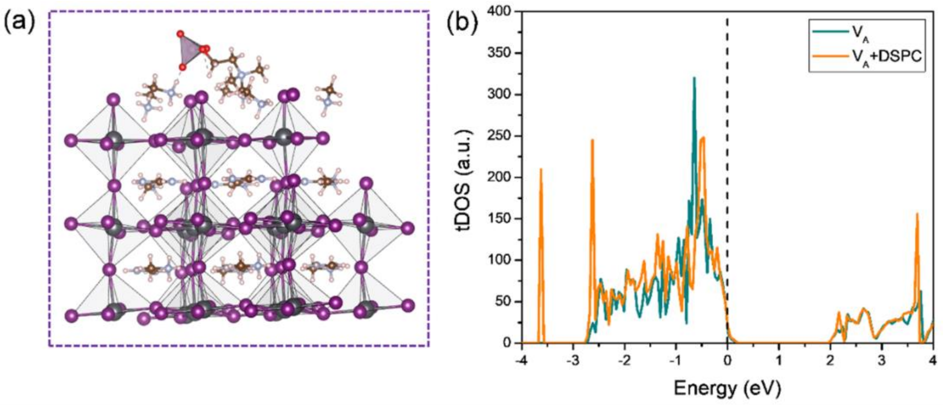

Fig. S15 a) Differential charge density distributions and Bader charge of perovskite interacting with DSPC. **b)** DFT calculated tDOS of V_A-containing perovskite and that with DSPC

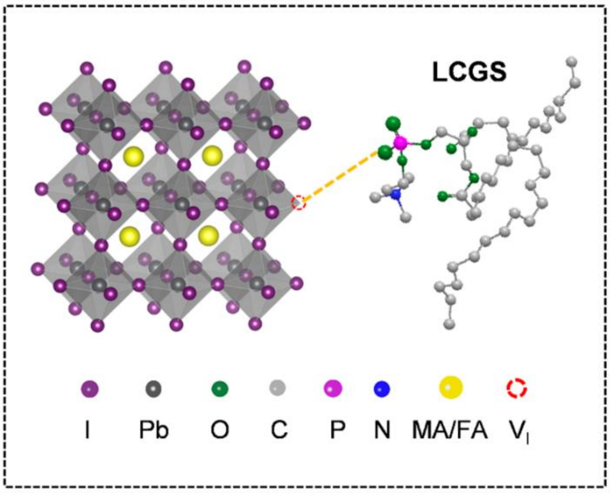

Fig. S16 Schematic diagram of V_I defect passivation in perovskite films by long-chain gemini surfactants (LCGS)

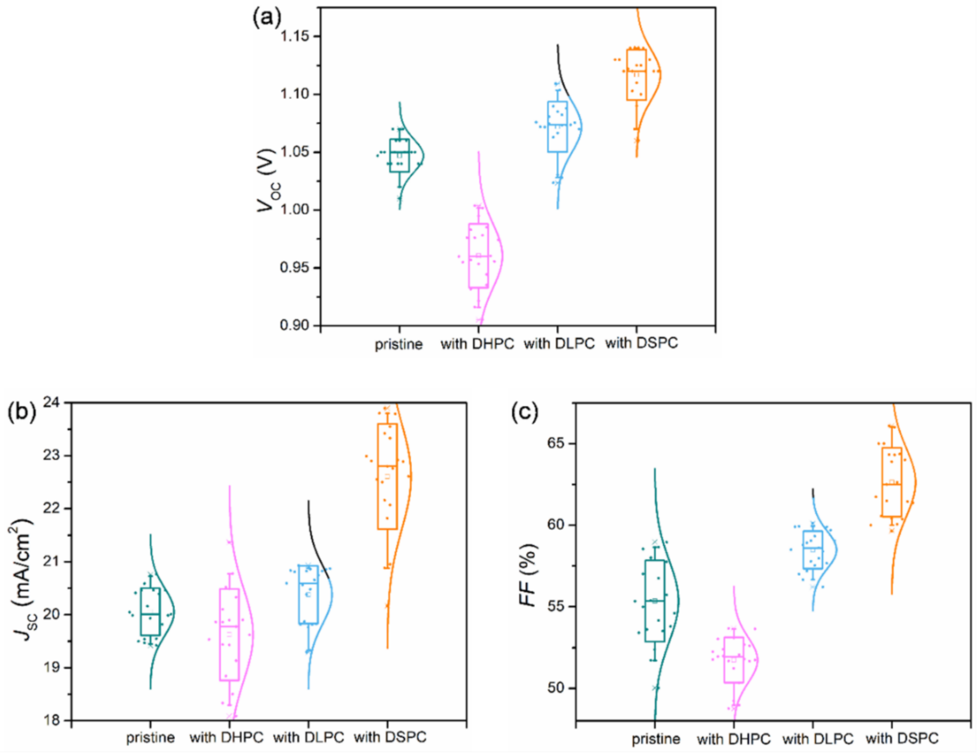

Fig. S17 Detailed statistics of performance parameters for the perovskite solar modules with and without the different surfactants: **a)** V_{oc}, **b)** J_{sc}, and **c)** *FF*

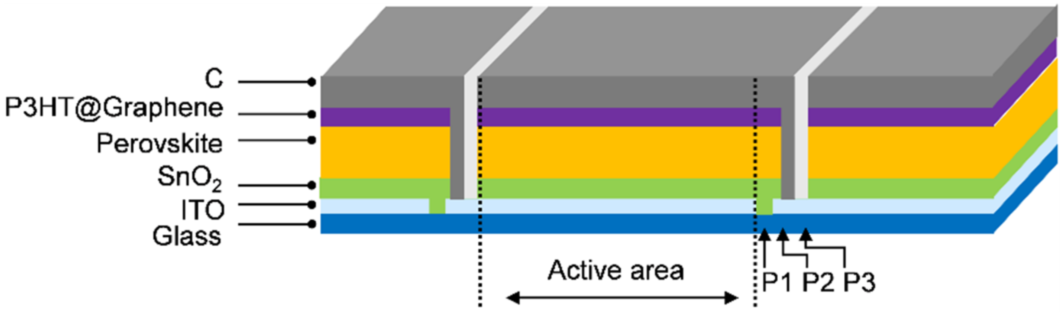

Fig. S18 Schematic of module structure with subcells connected in series

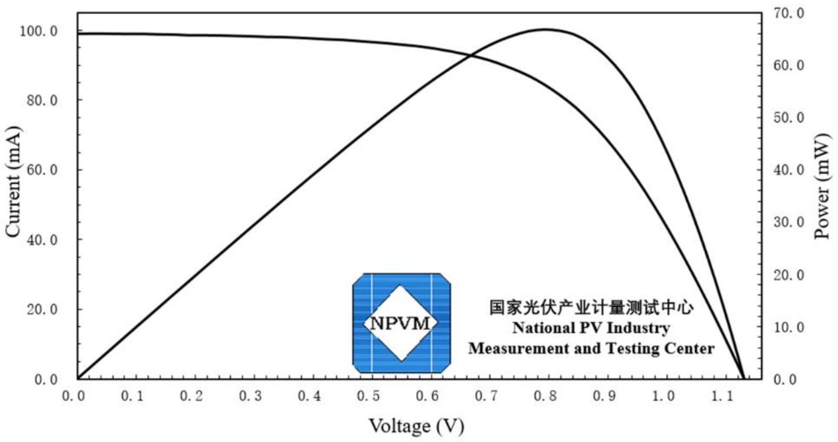

I_{sc} (mA)	V_{oc} (V)	I_{MPP} (mA)	V_{MPP} (V)	P_{MPP} (mW)	*FF* (%)	η (%)
99.00	1.131	84.32	0.7930	66.90	59.75	15.46

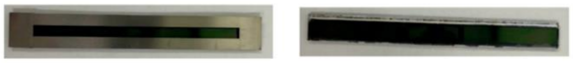

Fig. S19 The certified report of sub-cell in the fully blade coating carbon electrode based perovskite solar modules with DSPC doping, in which the active area of 4.32 cm^2 is determined by a mask hole

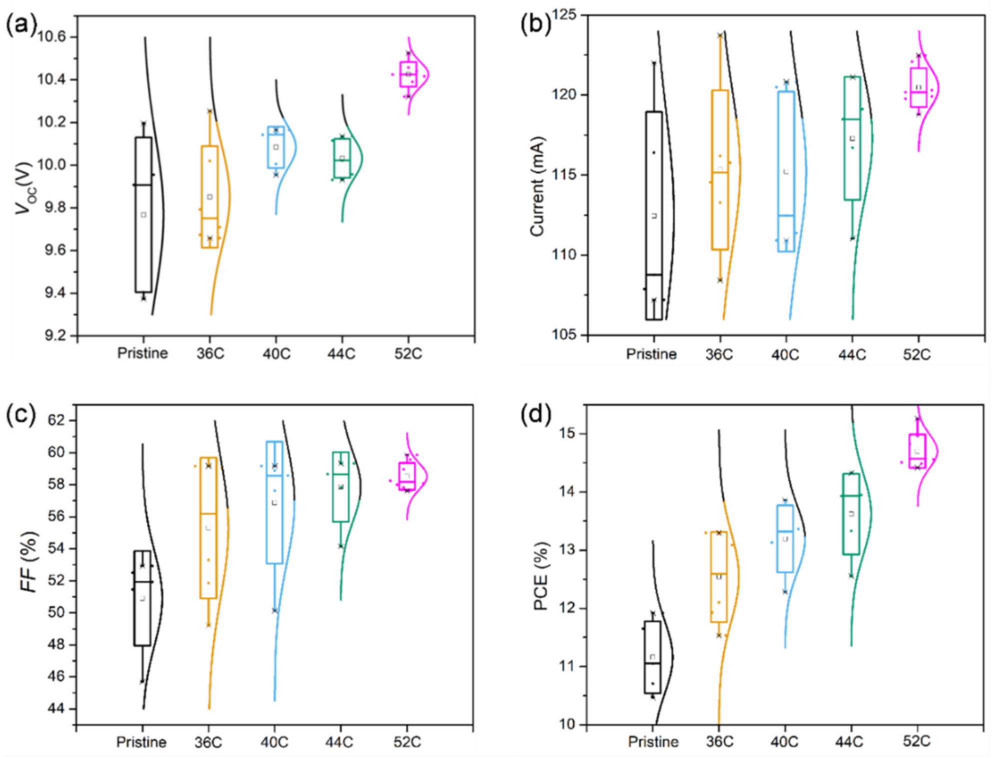

Fig. S20 Detailed statistics of performance parameters for the HTL-free perovskite solar modules with and without the different surfactants: **a)** V_{oc}, **b)** J_{sc}, **c)** *FF*, and **d)** PCE. DMPC(36C), DPPC(40C), DSPC(44C) and DDOCPC(52C) all exhibit positive effects, and DDOCPC has the best effect (**Fig.** S19). Carbon electrode perovskite solar modules were fabricated using the laser scribing and P4 edge cleaning procedures (see **Fig.** S21)

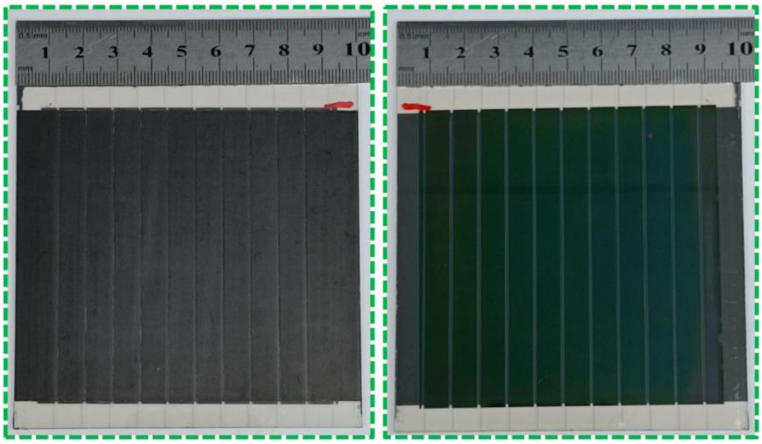

Fig. S21 The carbon electrode-side photo (left) and the glass-side photo (right) of the HTL-free carbon electrode perovskite solar module (ITO/SnO_2/Perovskite/C), which is entirely fabricated by the blade coating in ambient air. The module is fabricated by connecting 10 sub-cells in series by laser etching, such that the overlapping area through in different layers (active area) is 50 cm^2

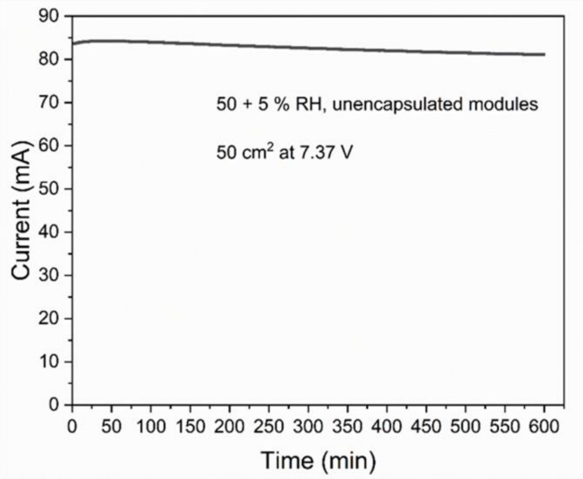

Fig. S22 Stabilized photocurrent output at the maximum power point (MPP) of the HTL-free carbon electrode based perovskite solar modules (ITO/SnO_2/Perovskite/C) with 10 sub-cells series connection after DSPC doping

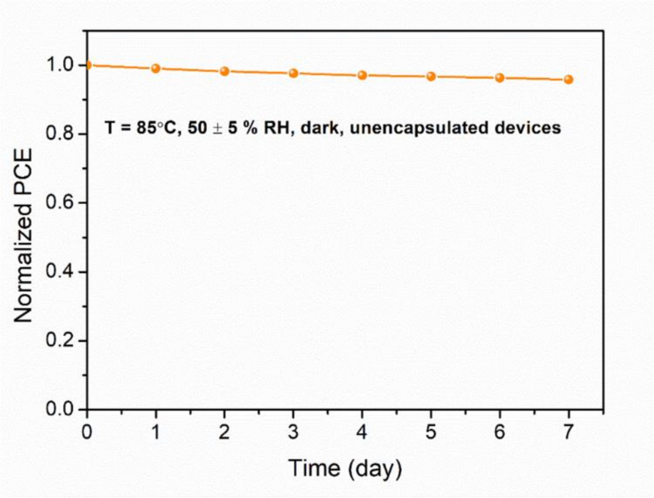

Fig. S23 The thermal stability (85°C) of the DSPC-PSCs

Supplementary References

[S1] G. Kresse, J. Furthmuller, Efficiency of ab-initio total energy calculations for metals and semiconductors using a plane-wave basis set. Comp. Mater. Sci. **6**(1), 15-50 (1996). https://doi.org/10.1016/0927-0256(96)00008-0

[S2] A. Binek, F.C. Hanusch, P. Docampo, T. Bein, Stabilization of the trigonal high-temperature phase of formamidinium lead iodide. J. Phys. Chem. Lett. **6**(7), 1249-1253 (2015). https://doi.org/10.1021/acs.jpclett.5b00380

[S3] J.P. Perdew, K. Burke, M. Ernzerhof, Generalized gradient approximation made simple. Phys. Rev. Lett. **78**(7), 1396-1396 (1997). https://doi.org/10.1103/PhysRevLett.78.1396

[S4] Y. Hu, S. Si, A. Mei, Y. Rong, H. Liu et al., Stable large-area (10 x 10 cm^2) printable mesoscopic perovskite module exceeding 10% efficiency. Sol. Rrl. **1**(2), 1600019 (2017). https://doi.org/10.1002/solr.201600019

[S5] A. Priyadarshi, L.J. Haur, P. Murray, D. Fu, S. Kulkarni et al., A large area (70 cm^2) monolithic perovskite solar module with a high efficiency and stability. Energy Environ. Sci. **9**(12), 3687-3692 (2016). https://doi.org/10.1039/c6ee02693a

[S6] G. Grancini, C. Roldan-Carmona, I. Zimmermann, E. Mosconi, X. Lee et al., One-year stable perovskite solar cells by 2d/3d interface engineering. Nat. commun. **8**, 15684 (2017). https://doi.org/10.1038/ncomms15684

[S7] M. Xu, W. Ji, Y. Sheng, Y. Wu, H. Cheng et al., Efficient triple-mesoscopic perovskite solar mini-modules fabricated with slot-die coating. Nano Energy **74**, 104842 (2020). https://doi.org/10.1016/j.nanoen.2020.104842

[S8] F. Yang, L. Dong, D. Jang, B. Saparov, K.C. Tam et al., Low temperature processed fully printed efficient planar structure carbon electrode perovskite solar cells and modules. Adv. Energy Mater. **11**(28), 2101219 (2021). https://doi.org/10.1002/aenm.202101219
